# Abscisic Acid Improves Linoleic Acid Accumulation Possibly by Promoting Expression of *EgFAD2* and Other Fatty Acid Biosynthesis Genes in Oil Palm Mesocarp

**DOI:** 10.3389/fpls.2021.748130

**Published:** 2021-12-03

**Authors:** Peng Shi, Wei Hua, Yin Min Htwe, Dapeng Zhang, Jun Li, Yong Wang

**Affiliations:** ^1^Oil Crops Research Institute, Chinese Academy of Agricultural Sciences, Key Laboratory of Biology and Genetic Improvement of Oil Crops, Ministry of Agriculture and Rural Affairs, Wuhan, China; ^2^Hainan Key Laboratory of Tropical Oil Crops Biology/Coconut Research Institute, Chinese Academy of Tropical Agricultural Sciences, Wenchang, China; ^3^Hainan Key Laboratory for Biosafety Monitoring and Molecular Breeding in Off-Season Reproduction Regions/SanYa Research Institute, Chinese Academy of Tropical Agricultural Sciences, Sanya, China

**Keywords:** oil palm, linoleic acid, abscisic acid, *FAD2*, ABRE motif

## Abstract

Abscisic acid plays an important role in fruit development. However, the effect of ABA on fatty acid biosynthesis in oil palm is still unknown. In this study, ABA treatments (CK, A1–A4) were applied to oil palm fruit at 16 WAP (weeks after pollination), and fatty acids in the mesocarp at 24 WAP were analyzed by GC-MS. Results showed that linoleic acid content under treatment A2 (20 μM ABA) was significantly higher (slightly increased by 8.33%) than the control. Therefore, mesocarp samples of A2, and the control at 16, 20, and 24 WAP was sampled for RNA-Seq. KEGG pathway enrichment analysis showed that 43 genes were differentially expressed in the fatty acid biosynthesis pathway, of which expression of *EgFAD2* (unigene 105050201) under 20 μM ABA treatment was 1.84-fold higher than in the control at 20 WAP. Further sequence analysis found that unigene 105050201 had more ABA-responsive elements (ABRE), complete conserved domains, and a C-terminal signaling motif among two *FAD2* copies. Furthermore, WGCNA and correlation analysis showed co-expression of *EgFAD2* (unigene 105050201) with transcription factors (TFs) (*WRI1*, *AP2-EREBP*, *bZIP*, *bHLH*, *C2C2-Dof*, *MYB*, *NAC*, and *WRKY*), ABA signaling genes (*PYR*, *PP2C*, *SnRK*, and *ABI5*), and other genes involved in fatty acid biosynthesis (*FATA*, *FATB*, *LACS*, *SAD*, *Oleosins*, and so on). These results indicated that ABA treatment promoted the expression of *FAD2* and other genes involved in fatty acid biosynthesis, which possibly resulted in the accumulation of linoleic acid. This study will be helpful for understanding the possible mechanisms through which ABA affects fatty acid biosynthesis and their accumulation in the mesocarp of oil palm.

## Introduction

Oil palm (*Elaeis guineensis*) is a valuable tropical oil crop, providing approximately 36% of edible oils globally (Zhou et al., 2020). Its ability to produce high oil yields has led to its cultivation in tropical regions throughout the world. The fruit oil content of oil palm is high (about 50%), while unsaturated fatty acid content (about 50%) is lower than for other oil crops, such as rapeseed and soybean. As unsaturated fatty acids have many health benefits, increasing the unsaturated fatty acid content of the mesocarp has important implications for improving palm oil quality.

The mesocarp is the most prolific oil production tissue in oil palm fruit; it mainly contains palmitic and oleic acids, and the fatty acid biosynthesis pathway and related genes have been already characterized (Dussert et al., 2013). Previous reports have shown that fatty acid biosynthesis is mainly involved in carbon chain extension and desaturation. Carbon chain extension takes place within plastids. In this process, fatty acids are synthesized from pyruvate, while pyruvate dehydrogenase complex (PDH), acetyl-CoA carboxylase (ACCase), malonyl-CoA (MAT), acyl carrier protein (ACP), ketoacyl-ACP synthase (KASI, KASII, and KASIII), ketoacyl-ACP reductase (KAR), hydroxyacyl-ACP dehydrase (HAD), enoyl-ACP reductase (EAR), stearate desaturase (SAD), and acyl-ACP thioesterase (FATA, FATB) participate in the process. Subsequently, 8:0–18:1 CoA are released to the endoplasmic reticulum by long-chain acyl-CoA synthetase (LACS). The 8:0–18:1 CoA is desaturated further by *ω*-6 desaturase (FAD2) and *ω*-3 desaturase FAD3 to concurrently synthesize triacylglycerol (TAG) with glycerol-3-phosphate (Glycerol-3-P). The enzymes glycerol-3-phosphate acyltransferase (GPAT), lysophosphatidic acid acyltransferase (LPAAT), phosphatidate phosphatase (PAP), diacylglycerol cholinephosphotransferase (CPT), phospholipid diacylglycerol acyltransferase (PDAT), 1-acylglycerol-3-phosphocholine acyltransferase (LPCAT), and diacylglycerol acyltransferase (DGAT) participate in this process. Finally, TAG accumulates in the cytoplasm with the oil formed by caleosin and oleosins proteins.

A few studies have investigated the effects of abscisic acid (ABA) on fatty acid biosynthesis in other organisms. Exogenous ABA increases levels of palmitic (C16:0) and stearic (C18:0) acids but decreases linoleic (C18:2) and *α*-linolenic (C18:3n3) acids in *Chlorella vulgaris* (Norlina et al., 2020). Exogenous ABA can enhance oleic (C18:1) and linoleic acid (C18:2) accumulation in developing Siberian apricot (*Prunus sibirica*) seeds, and *ABI3, SAD6, FAD2*, and *KCS1-like* showed upregulated expressions in ABA treatment compared with control (Huo et al., 2020); it can also enhance the linoleic acid and elaidic acid content in Cabernet Sauvignon (*Vitis vinifera* L.) grape berries (Ju et al., 2016). While studies on other plant species show that exogenous ABA influences linoleic acid biosynthesis, this process is still not well understood in oil palm. During mesocarp development, ABA remains at low levels at 12–14 weeks after pollination (WAP), while increasing sharply at 16–18 WAP and, thereafter, maintains a high level (Teh et al., 2014). Palmitic and oleic acid increased gradually from 10 to 23 WAP (weeks after pollination) in the mesocarp, while linoleic acid decreased gradually during these stages, with a sharp decline observed at 17 WAP. While ABA content increased, linoleic acid content declined simultaneously, suggesting that exogenous ABA plays an important role in fatty acid accumulation in oil palm mesocarp (Dussert et al., 2013). In order to confirm effects of exogenous ABA on fatty acid accumulation in oil palm mesocarp, we designed experiment of exogenous ABA treatment on oil palm fruit. Moreover, RNA-seq was also conducted to reveal a transcriptional mechanism that exogenous ABA regulated fatty acid accumulation.

## Materials and Methods

### Field Conditions and Materials

Five fruit bunches (according to one control and four ABA treatments, respectively) aged 16 WAP from a 6-year-old oil palm tree (fruit form: Tenera) were selected from the oil palm germplasm nursery in the Coconut Research Institute, Chinese Academy of Tropical Agricultural Sciences, Wenchang, China (19°31′50″N, 110°45′58″E) during April 2020. Oil palms were spaced at 8 m × 8 m and drip irrigated. Four ABA [S-(+)-ABA, Yuanye Bio-Technology Co., Ltd, Shanghai, China] concentrations (10, 20, 50, and 200 μM) and a control (CK; with double-distilled water treatment) were prepared (Table 1). At 9:00 h with calm wind, at 26–33°C, ABA treatments were applied by spraying on the surface of fruits for 3 days in a row in the field. Mesocarps from three oil palm fruits were collected randomly at 1 day before treatment (CK_1, A1_1, A2_1, A3_1, and A4_1), 4 weeks later (CK_3, A1_3, A2_3, A3_3, and A4_3), and 8 weeks later (CK_5, A1_5, A2_5, A3_5, and A4_5). Each sample was collected in three biological replicates and immediately frozen in liquid nitrogen. Thereafter, they were kept in a freezer at −80°C for further analyses (Table 1).

**TABLE 1:** Treatment and sampling information.

Treatment	CK	A1	A2	A3	A4
ABA concentration	0 μM	10 μM	20 μM	50 μM	200 μM
Fatty acid composition measure	CK_5(24 WAP)	A1_5(24 WAP)	A2_5(24 WAP)	A3_5(24 WAP)	A4_5(24 WAP)
RNA-Seq	CK_1(16WAP), CK_3(20WAP), CK_5(24WAP)		A2_1(16WAP), A2_3(20WAP), A2_5(24WAP)		

### Fatty Acid Analysis by GC-MS

Standard product configuration: 52 types of mixed standard solutions of fatty acid methyl ester were prepared with n-hexane to 0.5, 1, 5, 10, 25, 50, 100, 250, 500, 1,000, and 2,000 μg/ml concentration gradients. The concentration is the total concentration of each component.

#### Sample Pretreatment

Approximately 50 mg samples were mixed with 1 ml chloroform methanol (2:1) solution. The mixture was grounded in a high-throughput tissue grinder by shaking at 60 Hz for 1 min, and the process was repeated two times; subsequently, the mixture was subjected to ultrasound for 30 min at room temperature. Thereafter, the samples were centrifuged at 12,000 rpm at 4°C for 5 min. The supernatants were collected and mixed with 2 ml 1% sulfuric acid methanol, and vortexed for 1 min, and then followed by esterification in a water bath at 80°C for 30 min. After removal from the water bath, samples were allowed to cool and were then mixed thoroughly with 1 ml n-hexane and kept in room temperature for 5 min. To this mixture, 5 ml of ddH_2_O (4°C) was added, and centrifugation took place at 12,000 rpm at 4°C for 10 min. The supernatant (700 μl) was collected and mixed thoroughly with 100 mg anhydrous sodium sulfate powder to remove excess water and centrifuged at 12,000 rpm for 5 min. Then, 10 μl of supernatant was collected and mixed with 490 μl n-hexane. Thereafter, 300 μl of diluent was collected. A total of 15 μl l,500 ppm methyl salicylate, which was used as an internal standard, was mixed with the collected diluents, and 250 μl of supernatant was collected for GC-MS analysis.

#### GC-MS Analysis

GC-MS analysis was performed using a Thermo TG-FAME capillary column (50 m *0.25 mm ID *0.20 μm). The injection volume was 1 μl, and the split ratio was 8:1. The injection port temperature was 250°C, the ion source temperature was 230°C, the transmission line temperature was 250°C, and the quadrupole temperature was 150°C. The initial level of the programmed temperature was 80°C for 1 min; this subsequently rose to 160°C at 20°/min for 1.5 min; and 3°/min to 196° for 8.5 min. Finally, the temperature was raised to 250° at 20°/min for 3 min. Fatty acids were separated using helium as the carrier gas with a flow rate of 0.63 ml/min. The mass spectrometer was operated in the electron impact ionization (EI) mode at 70 eV.

### RNA Extraction and Transcriptome Sequencing

Ethanol precipitation protocol and CTAB-pBIOZOL reagent were used for the purification of total RNA from the plant tissue according to the manual instructions. Grind about 80 mg samples into powder with liquid nitrogen and transfer the powder into 1.5 ml preheated 65°C CTAB-pBIOZOL reagents. The samples were incubated by a Thermo mixer for 15 min at 65°C to permit the complete dissociation of nucleoprotein complexes. After centrifuging at 12,000 × g for 5 min at 4°C, the supernatant was added 400 μl of chloroform per 1.5 ml of CTAB-pBIOZOL reagent and was centrifuged at 12,000 × g for 10 min at 4°C. The supernatant was transferred to a new 2 ml tube that added 700 μl acidic phenol and 200 μl chloroform, followed by centrifuging 12,000 × g for 10 min at 4°C. The aqueous phase was added equal volume of an aqueous phase of chloroform and centrifuged at 12,000 × g for 10 min at 4°C. The supernatant was added equal volume of supernatant of isopropyl alcohol and placed at −20°C for 2 h for precipitation. After that, the mix was centrifuged at 12,000 × g for 20 min at 4°C and then removed the supernatant. After washing with 1 ml of 75% ethanol, the RNA pellet was air-dried in the biosafety cabinet and was dissolved by adding 50 μl of DEPC-treated water. Subsequently, total RNA was qualified and quantified using a Nano Drop and Agilent 2100 bioanalyzer (Thermo Fisher Scientific, MA, United States).

Oligo (dT)-attached magnetic beads were used to purified mRNA. Purified mRNA was fragmented into small pieces with a fragment buffer at appropriate temperature. Then, first-strand cDNA was generated using random hexamer-primed reverse transcription, followed by a second-strand cDNA synthesis. Afterward, A-Tailing Mix and RNA Index Adapters were added by incubating to end repair. The cDNA fragments obtained from a previous step were amplified by PCR, and products were purified by Ampure XP Beads, and then dissolved in EB solution. The products were validated on the Agilent Technologies 2100 bioanalyzer for quality control. The double-stranded PCR products from a previous step were heated, denatured, and circularized by the splint oligo sequence to get the final library. The single-strand circle DNA (ssCir DNA) was formatted as the final library. The final library was amplified with phi29 to make DNA nanoball (DNB), which had more than 300 copies of one molecular, DNBs were loaded into the patterned nanoarray, and single-end 50 bases reads were generated on a MGISEQ500 platform (BGI-Shenzhen, China).

### Transcriptome Data Processing, Reference Genome Selection, Differential Expression, and Functional Enrichment Analysis

The sequencing data were filtered with SOAPnuke v.1.4.0 and Trimmomatic v.0.36 (Li et al., 2008) by (1) removing reads containing a sequencing adapter; (2) removing reads whose the low-quality base ratio (base quality≤5) was>20%; (3) removing reads whose the unknown base (“N” base) ratio was>5%; afterward, clean reads were obtained and stored in a FASTQ format. The clean reads were mapped to the African oil palm genome^1^ using HISAT2 v.2.1.0 (Kim et al., 2015). Bowtie2 v.2.2.5 (Langmead and Salzberg, 2012) was used to align the clean reads with the reference-coding gene set, and then the expression levels of genes were calculated using RSEM v.1.2.8 (Li and Dewey, 2011). The heatmap was drawn using the “pheatmap” tool of the R package according to gene expression in different samples. Essentially, differential expression analysis was performed using the DESeq2 v.1.4.5 (Love et al., 2014) with *Q* ≤ 0.05. GO, and KEGG enrichment analysis of annotated differentially expressed genes was performed using the R function “Phyper” based on the hypergeometric test. The significant levels of terms and pathways were corrected using *Q*-value with a rigorous threshold (*Q* ≤ 0.05) according to Bonferroni.

### Statistical Analysis and Heatmap Drawing

Statistical analysis was performed to identify fatty acid compositions that were significant between treatments, using Fisher LSD in one-way ANOVA, at *p* < 0.05 or < 0.01. Correlation analysis was conducted using Origin 9.1. R package “pheatmap” was used for heatmap drawing.

### Network Construction and Performance

R package “WGCNA” was used to construct a WGCNA network, correlation coefficients were calculated using Origin 9.1, and Cytoscape 3.8 was used to perform networking using WGCNA and the correlation network file.

### *Cis*-Acting Element Prediction, Phylogenetic Tree Construction, and Protein Sequence Analysis

Promoter sequences used for *cis*-acting element prediction were downloaded from NCBI,^2^ and then the FASTA format of promoter sequences was submitted to the PlantCARE website^3^ for *cis*-acting element prediction. A phylogenetic tree was conducted in MEGA X using a maximum likelihood tree (Kumar et al., 2018). An alignment view of protein sequences was performed using GeneDoc software.

## Results

### Effects of Exogenous Abscisic Acid on Fatty Acid Composition in the Mesocarps of Oil Palm

**FIGURE 1:**
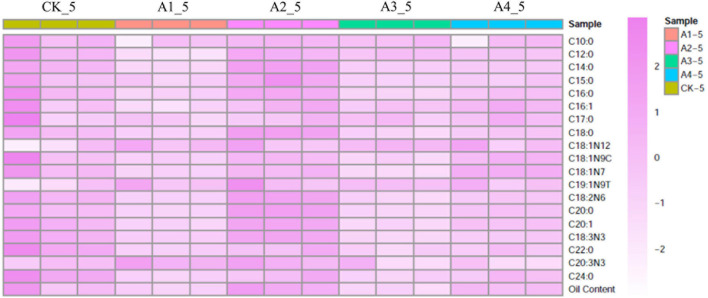
Heat map of fatty acid composition content after different exogenous ABA treatments. CK, treatment with 0 μM ABA; A1, treatment with 10 μM ABA; A2, treatment with 20 μM ABA; A3, treatment with 50 μM ABA; A4, treatment with 200 μM ABA. Each colored cell on the map corresponds to a concentration value.

To determine fatty acid content and composition, mesocarps of the five treatments at 24 WAP were measured by GC-MS (Table 1). No significant differences were observed on oil content between ABA-treated samples (A2 and A4) and the control (Figure 1 and Supplementary Table 1). The content of myristic acid, stearic acid, 10-transnonadecenoate, linoleic acid, arachidonic acid, behenic acid, and lignoceric acid increased significantly in A2 compared to CK, while content of main fatty acids components, such as palmitic acid and oleic acid, had no significant changes (Figure 1 and Supplementary Table 1). Among those fatty acids, linoleic acid increased by 8.33% in A2 compared to CK. Although the standard deviation of linoleic acid in CK_5 was big, linoleic acid was selected for the object of in-depth analysis because its content was highest in those fatty acids, and it could possess higher application value.

### Transcriptome Sequencing

Linoleic acid is reported to be an important unsaturated fatty acid in the oil palm mesocarp (Dussert et al., 2013). Therefore, A2 and CK samples at 16, 20, and 24 WAP were collected for RNA-seq analysis (Table 1). A total of 18 libraries were generated from the mesocarp of CK and A2 samples at 16, 20, and 24 WAP, with three biological replicates and produced 47.33–50.83 million raw reads (Table 2). After removing reads-containing adapters or poly-N and low-quality reads, the total number of clean reads per library was in the range of 41.95–43.48 million. Hierarchical indexing for spliced alignment of transcripts (HISAT) was used to compare clean reads to the reference genome^4^ using Bowite2. Among the short clean reads, 80.34–83.31% were aligned against the reference genome.

**TABLE 2:** Categorization and a summary of abundance of RNA-seq reads and genes in 18 libraries.

Sample	Total raw reads (M)	Total clean reads (M)	Total clean bases (Gb)	Clean reads Q20 (%)	Clean reads Q30 (%)	Clean reads ratio (%)	Total genome mapping (%)	Uniquely genome mapping (%)	Total gene mapping (%)	Uniquely gene mapping (%)
A2_1A	49.08	43.45	6.52	94.47	88.22	88.52	82.62	38.42	75.73	60.63
A2_1B	49.08	43.30	6.50	94.34	87.94	88.23	82.40	37.87	76.05	61.10
A2_1C	49.08	43.45	6.52	94.43	88.13	88.52	82.22	37.81	75.28	60.33
A2_3A	47.33	42.04	6.31	94.41	88.09	88.83	82.80	38.64	78.98	63.14
A2_3B	49.08	43.48	6.52	94.26	87.77	88.60	82.60	37.36	79.62	63.84
A2_3C	47.33	42.01	6.30	94.34	87.96	88.77	82.60	37.74	79.13	63.27
A2_5A	50.83	43.42	6.51	94.26	87.89	85.42	80.34	36.93	75.98	59.67
A2_5B	49.08	43.35	6.50	94.31	87.91	88.33	81.78	36.95	77.74	60.71
A2_5C	49.08	43.28	6.49	94.38	88.03	88.19	81.61	37.35	77.11	60.09
CK_1A	49.08	43.45	6.52	94.71	88.72	88.53	82.95	39.24	76.00	60.57
CK_1B	49.08	43.10	6.46	94.27	87.81	87.82	81.83	37.03	75.42	60.23
CK_1C	47.33	41.95	6.29	94.26	87.75	88.63	81.76	37.03	74.42	59.49
CK_3A	47.33	42.10	6.32	94.61	88.51	88.96	82.96	39.55	77.84	61.73
CK_3B	49.08	43.36	6.50	94.27	87.75	88.35	82.43	37.47	77.87	62.04
CK_3C	42.16	37.59	5.64	94.37	87.97	89.15	82.50	37.84	77.60	61.71
CK_5A	49.08	43.45	6.52	94.53	88.36	88.53	83.31	39.30	78.12	63.06
CK_5B	47.33	42.22	6.33	94.30	87.83	89.22	83.31	38.06	78.47	63.47
CK_5C	47.33	42.05	6.31	94.40	88.05	88.85	82.91	38.39	77.31	62.28

**FIGURE 2:**
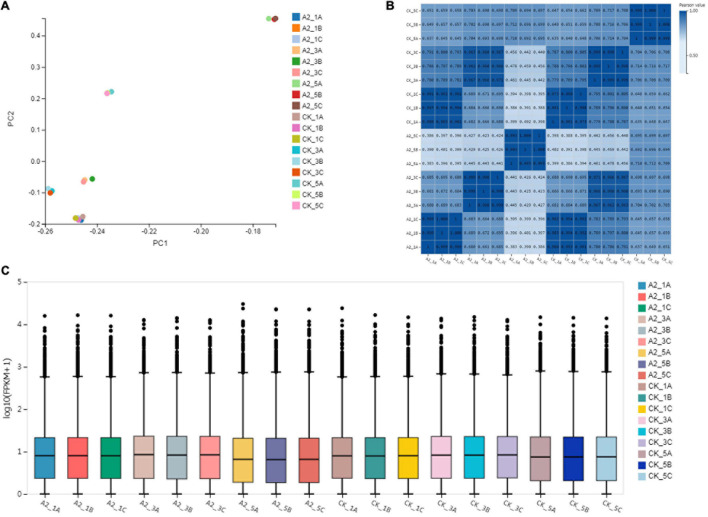
Transcriptome at 0, 4, and 8 weeks after treatment with 0 and 20 μM ABA. **(A)** Principal component analysis of six samples. **(B)** Pearson correlation coefficient of gene expression levels between samples. **(C)** Distribution of gene expression levels in each sample. The *x*-axis shows the sample name, and the *y*-axis shows log10 [FPKM (fragments per kilobase of exon model per million mapped reads) + 1]. The box plot for each region corresponds to five statistics (upper to lower, upper quartile, median, and lower quartile, lower limit, where the upper and lower limits do not take outliers into account).

Principal Component Analysis (PCA) showed that three biological replicates of each sample were well clustered. Two groups (CK_1 and A2_1) were clustered together, while the other four groups (CK_3, CK_5, A2_3, and A2_5) were distinguished from each other; in particular, A2_5 was found to be clearly distinguished from the remaining five groups (Figure 2A). To analyze the correlation of the samples, Pearson’s correlation coefficient was calculated based on gene expression levels. Results showed that CK_1 was highly correlated with A2_1, and CK_3 was highly correlated with A2_3. Meanwhile A2_5 was clearly separated from other samples (Figure 2B), which was consistent with the PCA analysis.

The distribution of gene expression levels in each sample was then performed, and the degree of dispersion of the data distribution was observed (Figure 2C). The median values of genes expression under A2_5 were significantly lower (*p* < 0.01) than A2_1 and A2_3, and the same to CK_5 (*p* < 0.01).

### Analysis of Differentially Expressed Genes

**FIGURE 3:**
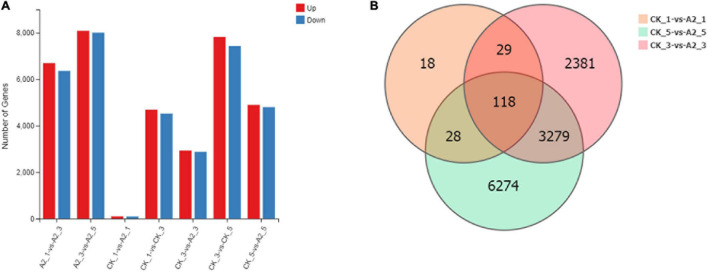
A summary of DEGs between CK and A2. **(A)** Number of DEGs. **(B)** The Venn diagram of DEGs between CK and A2.

A total of 193 DEGs were detected in CK_1 vs. A2_1, of which 98 DEGs were upregulated and 95 DEGs were downregulated. In total, 5,807 DEGs were identified in CK_3 vs. A2-3, of which 2,930 DEGs were upregulated and 2,877 DEGs were downregulated. In CK_5 vs. A2_5, a total of 9,699 genes were identified as DEGs, of which 4,898 DEGs were upregulated and 4,801 DEGs were downregulated. A total of 118 DEGs overlapped among the groups (Figures 3A,B). The lowest number of DEGs (193) was identified in CK_1 vs. A2_1, which was sampled before ABA treatment, while the number of DEGs identified in CK_3 vs. A2_3 and CK_5 vs. A2_5 was sharply increased. These results indicated that ABA treatment had a great impact on transcriptional changes in oil palm mesocarps.

DEGs were subjected to Gene Ontology (GO) analysis to further understand their functions. Among 5,807 DEGs in CK_3 vs. A2_3, 4,361 were classified into three main GO categories: biological process, cellular component, and molecular function. The main biological processes represented were cellular process (1,309), metabolic process (1,144), and biological regulation (415). Major cellular component included membrane (1,386), cell (1,332), and membrane part (1,322). The main molecular functions were binding (2,246), catalytic activity (2,133), and transporter activity (260) (Supplementary Figure 1A and Supplementary Table 2). The top 20 GO enrichments were further analyzed, and results showed that the fatty acid biosynthetic process under the biological process was observed in these GO terms (Supplementary Figure 1B and Supplementary Table 3).

To identify significantly enriched pathways in CK_3 vs. A2_3, pathway annotation was conducted *via* the Kyoto Encyclopedia of Genes and Genomes (KEGG). In total, 2,763 DEGs were annotated in KEGG pathways; of which, 303, 263, and 271 DEGs were enriched in signal transduction, transcription, and lipid metabolism, respectively (Supplementary Figure 1C and Supplementary Table 4). We further analyzed the top 20 enriched pathways and observed that fatty acid biosynthesis and fatty acid metabolism were represented (Supplementary Figure 1D and Supplementary Table 4). Several pathways related to fatty acids were annotated: pyruvate metabolism (75), fatty acid metabolism (55), and fatty acid biosynthesis (43).

In CK_5 vs. A2_5, 7,227 of 9,699 DEGs were annotated in GO categories (Supplementary Figure 2A and Supplementary Table 5): Cellular process (2,122), metabolic process (1,842), and biological regulation (673) were represented as major categories under the biological process. Under the cellular component, membrane (2,354), membrane part (2,239), and cell (2,161) were the most highly represented categories. Major categories in molecular function were catalytic activity (3,536), binding (3,529), and transporter activity (432). When the top 20 GO categories were further analyzed, it was observed that cytoplasm (1,097), cytoplasmic part (882), and small molecule metabolic process (488) were the most highly represented categories (Supplementary Figure 2B and Supplementary Table 6). Significantly enriched KEGG pathways in CK_5 vs. A2_5 were further analyzed. In total, 4,453 DEGs were enriched in KEGG pathways (Supplementary Figure 2C and Supplementary Table 7). Similar to CK_3 vs. A2_3, fatty acid metabolism (73) and fatty acid biosynthesis (50) were represented as the top 20 KEGG pathways (Supplementary Figure 2D and Supplementary Table 7).

### *EgFAD2* Upregulation Possibly Results in Increase of Linoleic Acid Biosynthesis

**FIGURE 4:**
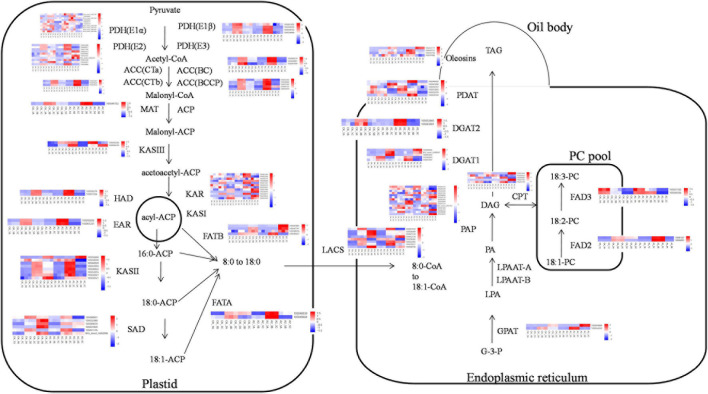
The transcriptional model of fatty acid biosynthesis in developing oil palm mesocarp. The 18 squares in each horizontal row correspond to three replicates of three stages (16 WAP, 20 WAP, and 24 WAP) in CK and A2. G-3-P, glycerol-3-phosphate; LPA, 1-acylglycerol-3P; PA, phosphatidic acid; DAG, 1,2-diacylglycerol; and TAG, triacylglycerol.

As exogenous ABA has a distinct impact on fatty acid accumulation, we further focused on the genes involved in lipid biosynthesis and ABA signaling pathways to identify possible mechanisms. A total of 14, 31, 520, 190 unigenes were, respectively, identified as ABA biosynthesis and metabolism, ABA signal transduction, TFs that could be related with ABA and fatty acid biosynthesis, and fatty acid biosynthesis in these transcriptome data (Supplementary Table 10). Among these unigenes, genes, such as *KAR*, *FAD2*, and *SAD*, were highly expressed with>1,000 FPKM value, suggesting that these genes may play an important role in fatty acid synthesis. Most upregulated genes in the fatty acid biosynthesis pathway were expressed in A2_3 compared with CK_3, such as *PDH(E1*α*)*, *PDH(E1*β*)*, *PDH(E2)*, *ACC(CTb)*, *ACC(BC)*, *ACC(BCCP)*, *MAT*, *KASIII*, *HAD*, *EAR*, *KAR*, *KASII*, *FATA*, *FATB*, *LACS*, *GPAT*, *FAD2*, *FAD3*, *DGAT2*, *PDAT*, and *Oleosins* (Figure 4). Meanwhile some genes were upregulated in A2_5 compared with CK_5, such as *PDH(E2), KASIII, KAR, KASII, FATB, GPAT, PAP, CPT, DGAT1*, and *Oleosins* (Figure 4). In addition, several genes involved in ABA signal transduction and TFs were also differentially expressed in A2_3_vs. CK_3 and A2_5_vs. CK_5, such as *PYR, PP2C, SnRK, ABI5, AP2-EREBP, bZIP, C2C2-Dof*, and *MYB* (Supplementary Table 8). In addition, exogenous ABA also affected the expression level of genes involved in ABA biosynthesis and metabolism (Supplementary Table 8). The expression level of some genes involved in ABA biosynthesis reduced after exogenous ABA treatment, such as *DXS, DXR*, and *ZEP*, while *PSY, PDS*, and *NCED* rose. Especially, the expression level of gene (*CYP707A*) involved in ABA metabolism was significantly (*p*-value < 0.01) higher in A2_5 than CK_5.

**FIGURE 5:**
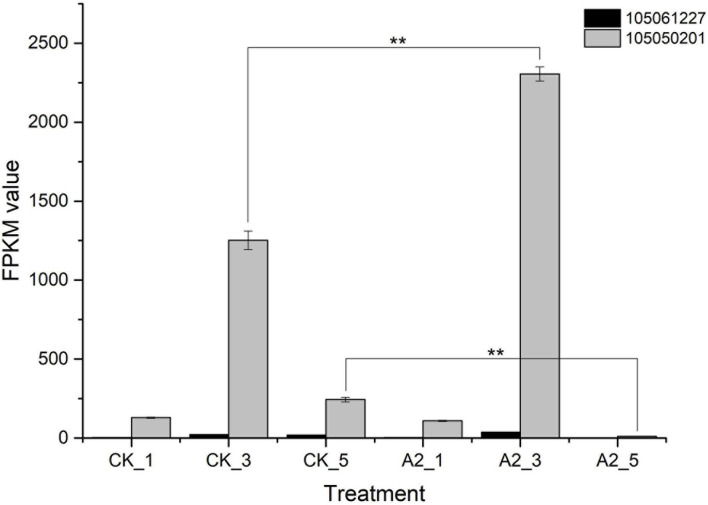
*FAD2* expression in CK and A2. **Represents significance at *p* < 0.01. CK_1: 16 WAP with 0 μM ABA; CK_3: 20 WAP with 0 μM ABA; CK_5: 24 WAP with 0 μM ABA; A2_1: 16 WAP with 20 μM ABA; A2_3: 20 WAP with 20 μM ABA; A2_5: 24 WAP with 20 μM ABA.

Moreover, the content of other major fatty acid components did not increase significantly instead of linoleic acid, so we focused on linoleic acid (Supplementary Table 1). In the fatty acid biosynthesis pathway (Figure 4), *FAD2* is the key gene-catalyzing oleic acid (C18:1) to linoleic acid (C18:2) (Dussert et al., 2013). In this study, two genes encoding *FAD2* (gene ID:105061227, 105050201) were differentially expressed, of which the expression level of unigene 105050201 under A2_3 was significantly higher than for CK_3, the same to A2_5 vs. CK_5 (Figure 5), suggesting that exogenous ABA treatment can lead to the increased expression of *FAD2*. This result indicated the *FAD2* expression level was raised by exogenous ABA.

In order to investigate whether *FAD2* could respond to an ABA signal, the abscisic acid responsiveness element (ABRE) was predicted using a promoter and the PlantCARE online tool. Results showed that the promoter of *FAD2* had an ABRE *cis*-acting element. Unigene 105050201 had the highest expression level and had six ABRE motifs of the type “ACGTG” (Table 3). Similarly, unigene 105061227 had eight ABRE motifs.

**TABLE 3:** ABRE cis-acting element prediction of *FAD2.*

Gene ID	Sequence	Position	Matrix Score	Strand	Organism
LOC105050201	GCAACGTGTC	755	9	+	*Hordeum vulgare*
	CACGTG	757	6	+	*Arabidopsis thaliana*
	ACGTG	758	5	+	*Arabidopsis thaliana*
	CACGTG	807	6	+	*Arabidopsis thaliana*
	ACGTG	808	5	+	*Arabidopsis thaliana*
	ACGTG	1,699	5	+	*Arabidopsis thaliana*
LOC105061227	CACGTG	183	6	+	*Arabidopsis thaliana*
	ACGTG	184	5	+	*Arabidopsis thaliana*
	GCAACGTGTC	376	9	+	*Hordeum vulgare*
	AACCCGG	705	7	-	*Arabidopsis thaliana*
	CACGTG	1,776	6	-	*Arabidopsis thaliana*
	ACGTG	1,777	5	+	*Arabidopsis thaliana*
	CACGTG	2,767	6	-	*Arabidopsis thaliana*
	ACGTG	2,768	5	+	*Arabidopsis thaliana*

**FIGURE 6:**
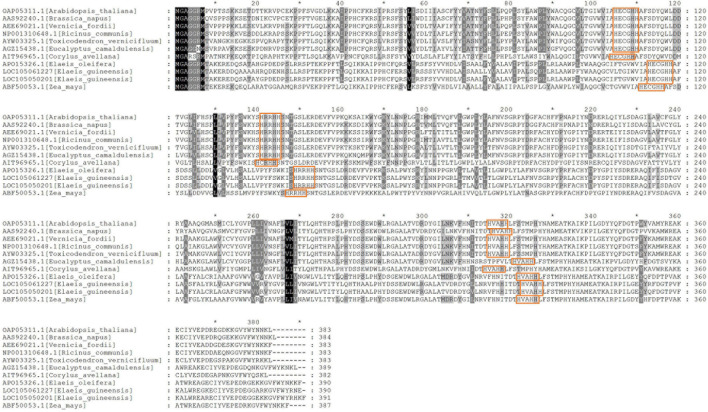
An alignment view of two FAD2 copies. Red blocks represent histidine-rich domains.

The sequence alignment analysis showed that AtFAD2 had 383 amino acid residues, and unigenes 105050201 and 105061227 had 390 and 391 residues, respectively (Figure 6). Three highly conserved histidine-rich motifs at EgFAD2 were included (HECGHH, HRRHH, and HVAHH). Meanwhile, unigenes 105050201 and 105061227 had complete histidine-rich motifs, and the lengths of protein sequences were similar to those of other species. The AtFAD2 protein can bind to endoplasmic reticulum because of the C-terminal signaling motif (YNNKL). Results showed that the C-terminal of unigene 105050201 was “YRHKF,” and, for unigene 105061227, it was “YRNE,” indicating that unigene 105050201 may also bind to endoplasmic reticulum.

In addition, the phylogenetic tree showed that unigenes 105061227 and 105050201 clustered together with American oil palm and maize FAD2. Results indicated that unigenes 105061227 and 105050201 had similar protein sequences (Supplementary Figure 3).

In conclusion, the promoter of unigene 105061227 had many ABRE motifs, and the protein sequence contained a complete histidine-rich domain, but the C-terminal lacked one resident compared to other organisms. A large number of ABRE motifs were found in the promoter region of unigene 105050201, and protein sequences contained complete histidine-rich domains, suggesting that expression of unigene 105050201 was upregulated in response to the ABA signal and thereby promoted linoleic acid synthesis.

### Correlation Analysis Among *FAD2*, Transcription Factors, and Genes Involved in Fatty Acid Biosynthesis

To obtain a comprehensive understanding of genes that possibly play an important role in linoleic acid biosynthesis in oil palm during ABA treatments, weighted gene co-expression network analysis (WGCNA) was performed. After filtering the genes with a low expression (FPKM < 0.05), 1,348 genes were selected for WGCNA. Co-expression networks were constructed based on pairwise correlations of gene expression across all samples.

This analysis identified 11 distinct modules (Supplementary Figure 4A). The 11 modules were correlated with distinct samples according to sample-specific expression profiles. A total of 28 genes involved in fatty acid biosynthesis and ABA signal transduction were found in the WGCNA network (Supplementary Figure 4B). A total of 47 genes, including TFs, such as *MYB*, *bZIP*, and *bHLH*, were significantly correlated with *PYR* (Supplementary Figure 4C and Supplementary Table 9). These TFs were reported to play a role in response to ABA signaling (Cutler et al., 2010). *WRI1* and *FAD2* were in a small network (Supplementary Figure 4D). Moreover, promoters of *PYR*, *PP2C*, *SnRK*, *ABI5*, *WRI1*, AP2-EREBP, *bZIP*, *C2C2-Dof*, *MYB*, and *NAC* were also found to possess ABRE motifs (Supplementary File 1 and Supplementary Table 10).

**FIGURE 7:**
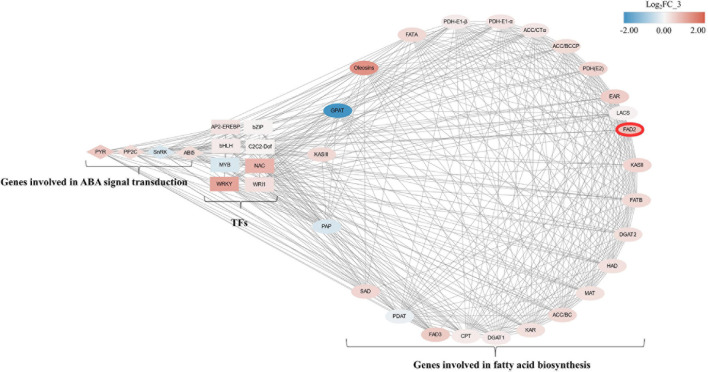
Correlation network of genes involving fatty acid biosynthesis, ABA-signaling transduction, and transcriptional factors between CK and A2 during mesocarp development. Ellipses, diamonds, and rectangles, respectively, represent genes involving fatty acid biosynthesis, ABA-signaling transduction, and transcriptional factors. Node fill color is according to log_2_FC of FPKM value in CK_3 vs. A2_3 from blue to red; TFs, transcriptional factors; PYR, pyrabactin resistance; PP2C, type 2C protein phosphatases; SnRK, Snf1-related protein kinase; ABI5, ABA insensitive 5; AP2-EREBP, APETALA2-ethylene responsive element-binding protein; bZIP, basic leucine zipper; bHLH, basic helix loop helix; C2C2-Dof, downstream of FGFR; MYB, myeloblastosis; NAC, nascent polypeptide-associated complex; WRKY, WRKY transcriptional factor; WRI1, WRINKLED1; KASIII, 3-ketoacyl-ACP synthase III; GPAT, glycerol-3-phosphate acyltransferase; FATA, fatty acid acyl-ACP thioesterase A; PDH-E1-β, subunit b of E1 component of PDH complex; PDH-E1-α, subunit α of E1 component of PDH complex; ACC/CTα, carboxyltransferase a-subunit of heteromeric ACCase; ACC/BCCP, biotin carboxyl-carrier protein of heteromeric ACCase; PDH(E2), E2 component of PDH complex; EAR, enoyl-ACP reductase; LACS, long-chain acyl-CoA synthetase; FAD2, ω-6 desaturase; KASII, 3-ketoacyl-ACP synthase II; FATB, fatty acid acyl-ACP thioesterase B; DGAT2, diacylglycerol acyltransferase 2, HAD, 3-hydroxyacyl-ACP dehydratase; MAT, malonyl-CoA: ACP transacylase; ACC/BC, biotin carboxylase subunit of heteromeric acetyl-CoA carboxylase; KAR, 3-ketoacyl-ACP reductase; DGAT1, diacylglycerol acyltransferase 1; CPT, CDP-choline: DAG cholinephosphotransferase; FAD3, ω-3 desaturase; PDAT, phospholipid: diacylglycerol acyltransferase; SAD, stearoyl-ACP desaturase; PAP, phosphatic acid phosphohydrolase.

In order to clarify the relationship between FAD2 and genes involved in fatty acid biosynthesis, ABA signaling transduction, and TFs, a total of 29 genes involved in fatty acid biosynthesis, ABA signaling transduction, and eight TFs were selected according to FPKM value > 10 for correlation analysis. The pairs significantly correlated (*p* < 0.05) were included in the network. *FAD2* (unigene 105050201) was found to be significantly correlated with *SnRK*, *ABI5*, *C2C2-Dof*, and *WRI1*; and 19 genes were involved in fatty acid biosynthesis (Figure 7).

## Discussion

Polyunsaturated fatty acids are essential for human health but cannot be synthesized by the human body; however, they can be supplied *via* edible vegetable oils. Palm oil plants produce the largest supply of edible vegetable oil in the world. It would, therefore, be of great benefit to improve the unsaturated fatty acid content in oil palm mesocarps, which composes around 50%. Research shows that palmitic acid content is around 44% in the mesocarp, with oleic acid at 40% and linoleic acid at 10% (Barcelos et al., 2015). So, there is a large potential for increasing linoleic acid levels. While previous studies on other plant species show that ABA could enhance the accumulation of linoleic acid (Ju et al., 2016; Huo et al., 2020; Norlina et al., 2020), the effect of ABA on oil palm mesocarp is still unknown. Results in this study showed 20 μM ABA was found to significantly promote linoleic acid content in mesocarp compared to an untreated control. A similar effect was also observed in Cabernet Sauvignon (*Vitis vinifera* L.) grape skins when 200 and 600 mg⋅L^–1^ ABA was applied to the surface of grape berries (Ju et al., 2016). In order to investigate transcriptional changes between control and ABA-treated samples, RNA-Seq analysis was conducted.

Compared with CK_1 vs. A2_1, abundant DEGs were, respectively, identified in CK_3 vs. A2_3 and CK_5 vs. A2_5. Results indicated that the spraying of ABA altered the transcriptional levels of a large number of genes in oil palm mesocarps. Moreover, DEGs analysis showed that many genes involved in ABA signal transduction, TFs, and fatty acid biosynthesis were differentially expressed in CK_3 vs. A2_3 and CK_5 vs. A2_5, such as *PYR*, *PP2C*, *SnRK*, *ABI5*, *AP2-EREBP*, *bZIP*, *C2C2-Dof*, *MYB*, *WRI1*, *FATA*, *FATB*, *LACS*, *GPAT*, *FAD2*, *DGAT2*, *PDAT*, and *Oleosins*. At the same time, the expression level of genes involved in ABA biosynthesis and metabolism was also affected by exogenous ABA. The expression level of some genes involved in ABA biosynthesis was up and down, but the expression level of gene involved in ABA metabolism was up. It indicated that exogenous ABA could affect ABA biosynthesis and promote endogenous ABA metabolism in mesocarp. There was no obvious change pattern for genes involved in ABA biosynthesis, possibly because of a large number of endogenous ABA synthesis during development and ripening of fruits (Teh et al., 2014).

The content of linoleic acid increased significantly after exogenous ABA treatment, while other major fatty acid components almost unchanged. It indicated that linoleic acid biosynthesis was affected by exogenous ABA, so genes involved in linoleic acid biosynthesis were emphatically analyzed. FAD2 is reported as a key enzyme-controlling linoleic acid biosynthesis (Dussert et al., 2013; He et al., 2020; Wang et al., 2021); thus, two unigenes annotated as *FAD2* were analyzed further. Of these two unigenes, expression levels of unigene 105050201 under A2_3 and A2_5 were significantly higher than CK_3 and CK_5, respectively, while unigene 105061227 still kept a low expression level. These results indicated that unigene 105050201 could respond to exogenous ABA. ABRE is an important element response to the ABA signal; ABA-responsive elements motif “ACGTC” was detected in the *FAD2* promoter region, and *FAD2* expression is known to be regulated by ABA (Kim et al., 2006; Xiao et al., 2014; Dar et al., 2017). Genes encoding the FAD2 enzyme contain eight conserved histidine residues in three clusters (HXXXH, HXXHH, and HXXHH) (Okuley et al., 1994). In the current study, similar ABRE motifs and histidine-rich motifs were found in unigene 105050201. Moreover, only unigene 105050201 had similar residents in the C-terminal as found in other organisms (Dehghan and Yarizade, 2014), indicating that unigene 105050201 may play a major role in promoting linoleic acid accumulation *via* response to the ABA signal.

In order to understand the possible mechanism of ABA-regulating linoleic acid biosynthesis, genes involved in ABA signal transduction and related TFs were further analyzed. Previous study found that ABA-signaling genes, such as *PYR*, *PP2C*, *SnRK*, and ABI5, could activate some TFs (*bZIP*, *MYB*, *MYC*, *AP2*, *NAC*, *WRKY*, and *bHLH*); these TFs subsequently activate the expression of downstream protein-coding genes (Kim et al., 2007; Cutler et al., 2010; Aleman et al., 2016). Our results showed many genes included in fatty acid biosynthesis and ABA signaling, such as *PYR*, *ABI5*, and *WRI1* were increased significantly after ABA treatment, indicating their response to ABA (Supplementary Table 10).

A previous study shows that fatty acid biosynthesis is subjected to phytohormones, such as ABA, auxin, and jasmonic acid (JA) (Shahid et al., 2019). Linoleic acid, one of the most common unsaturated fatty acids, acts as a precursor of jasmonates and a regulator of stress signaling (He and Ding, 2020). Moreover, jasmonic acid also crosstalks with the ABA signaling pathway (Aleman et al., 2016; Yu et al., 2021). Previous research found that TFs play an important role in regulating fatty acid biosynthesis. For example, *NF-YA3*, *NF-YC2*, and *ABI5* directly activate *WRI1* and regulate fatty acid biosynthesis (Yeap et al., 2017). Furthermore, *ABI5* is a *bZIP* type of TF involved in ABA signal transduction. As a member of *AP2-EREBP*, *WRI1* also plays a key regulatory role in fatty acid biosynthesis within the oil palm mesocarp. In the current study, *ABI5* and *WRI1* were upregulated after ABA treatment. Moreover, ABRE motifs were detected in promoters of *ABI5*, *WRI1*, and *FAD2*, suggesting that these genes could respond to the ABA signal.

*FAD2* expression has a high correlation with transcriptional factors, such as *WRI1*, *Dof*, and *ABI5* (Deng et al., 2019; He and Ding, 2020). WGCNA is an effective tool for exploring relationships across genes involved in fatty acid biosynthesis (Niu et al., 2020; Ma et al., 2021). Correlation analysis also showed that *EgFAD2* is co-expressed with these TFs, with several genes being involved in ABA signal transduction and fatty acid biosynthesis. These results suggested that the expression of *EgFAD2* was upregulated as a response to ABA signaling, possibly resulting in increased accumulation of linoleic acid.

**FIGURE 8:**
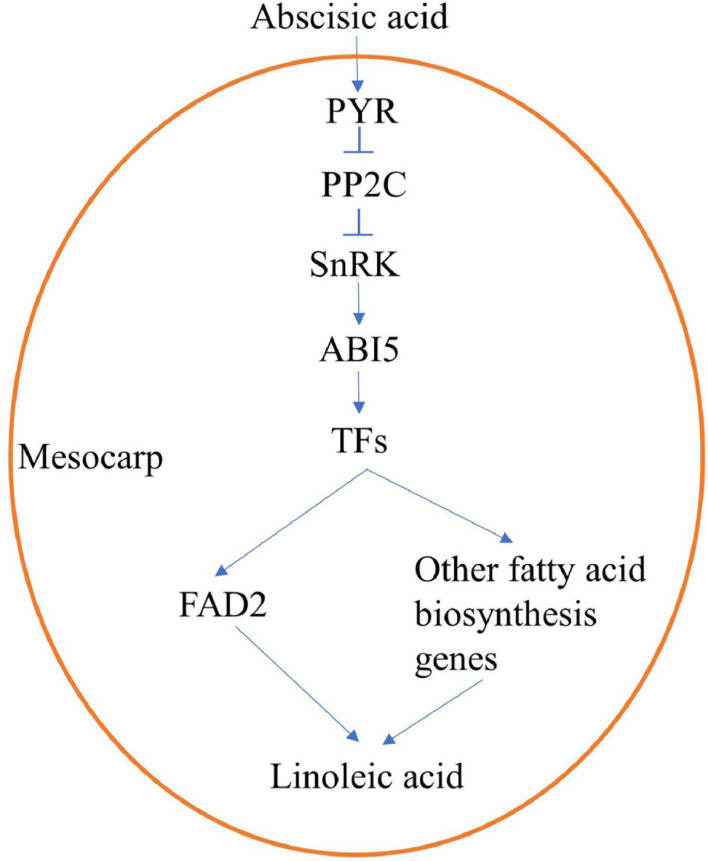
An speculative regulation mechanism of ABA-promoting linoleic acid biosynthesis in oil palm mesocarp.

Under exogenous ABA treatment, content of linoleic acid increased and expression of ABA-signaling genes, TFs, and fatty acids biosynthesis genes upregulated. Therefore, we speculated that ABA activated TFs through ABA signal transduction (*PYR*, *PP2C*, *SnRK*, and *ABI5*), and then promoted expression levels of *FAD2* or/and other fatty acids biosynthesis genes, resulting in increased accumulation of linoleic acid (Figure 8). Needless to say, further research is still required regarding the molecular mechanism by which ABA regulates linoleic acid biosynthesis; this can be extended from the current findings. Further experiments should be conducted to identify the upstream regulation factors of *FAD2* or other fatty acids biosynthesis genes, such as electrophoretic mobility shift assays, yeast one-hybrid and yeast two-hybrid assays, and ChIP-seq.

## Data Availability Statement

The datasets presented in this study can be found in online repositories. The names of the repository/repositories and accession number(s) can be found at: https://www.ncbi.nlm.nih.gov/, PRJNA719677.

## Author Contributions

PS and WH directed the project and wrote the manuscript. DZ and YH performed data analysis and revised the manuscript. YW and JL supervised this work and revised the manuscript. All authors read and approved the final manuscript.

## Conflict of Interest

The authors declare that the research was conducted in the absence of any commercial or financial relationships that could be construed as a potential conflict of interest.

## Publisher’s Note

All claims expressed in this article are solely those of the authors and do not necessarily represent those of their affiliated organizations, or those of the publisher, the editors and the reviewers. Any product that may be evaluated in this article, or claim that may be made by its manufacturer, is not guaranteed or endorsed by the publisher.

## Footnotes

1
https://www.ncbi.nlm.nih.gov/assembly/GCF_000442705.1

2
https://www.ncbi.nlm.nih.gov/

3
http://bioinformatics.psb.ugent.be/webtools/plantcare/html/

4
https://www.ncbi.nlm.nih.gov/genome/2669
