# Supplementary material for: Abscisic Acid Improves Linoleic Acid Accumulation Possibly by Promoting Expression of EgFAD2 and Other Fatty Acid Biosynthesis Genes in Oil Palm Mesocarp
Source: Front Plant Sci. 2021 Dec 3;12:748130. doi: 10.3389/fpls.2021.748130 (PMC8678531; doi:10.3389/fpls.2021.748130)
Supplement: Supplementary file 1 [file Data_Sheet_1.docx]

Supplementary File 1 Promoters of genes involved in ABA signal transduction, TFs, and FAD2

>3 kb from ‘ATG’ of LOC105050201

1 tgatgaaata attaaattta agtttgaatt tttatcttat ttaataaata aataaatcag

61 atttatatca ataaaatttt tatctatctc gaattcgaat cgatctatat cttatacgat

121 ccaatttgat ggttatctct atgccaactt gattgtaagc attccgcctg atccgagccc

181 gtgggtgata ggtcatcggt gttacatgca gcctgcttga cactaaaatg gacgcatcac

241 aaaaatacaa ataagtccct ttgtaacgaa agtggaatat cttattatta tgtgaatatt

301 cgaatatttg aaaagaagcc tctcagcctg atttattttt attacatctt aataaaaaaa

361 aaaaactcgc gcagaagaca cgacaaggcg gcacgagcaa agatacttac agcacaccct

421 aataaccaaa ttaccaccct acccttacac aagatgatca attggagggg caaaatggtc

481 tttgaggcac ggcgtggggc attggagggg caaaatggtc ttttggtcat ttgagtggct

541 tctccgtcaa atatttaaag gtgagggtta atttggtcat tttggcacaa aatcccagat

601 tttttttcct tctagtgcga actctggccg tccaaatgcg ttatcactaa cgggccacga

661 acggcggaga tgtcgtcagt tttgggaccg tgtcgcatgc ggccttcctc gtagcttacg

721 cctgctgttc ggtcagatca tccaatcgga tttggccacg tgtctcgaga tacgattcgc

781 aagagtgcgg ttccgtctcc gccgttcacg tgggcccagg ggcgggacca tcacccgtcg

841 cggggaaagc ttaaataccg catcccctgg gggtgagagg aaggcctccg tggacggcga

901 aaggaggggg aagcgggtgc gagagactaa atatatatta tatttttata tgtttggtct

961 cttctctcgc tctccaccct ctctgtgatt tacgcggcca tttttttttc tcaggtacga

1021 acatttcttc ttcatcccct ttttttcttt tttttttcct gttcgatgag tggatgttgt

1081 tgtgtgcatc gaaatcaatc tcttagtttt gggacttggt tcggtttgat cttgtggtta

1141 tcttcttcta cgatctgttt ctcgatggaa atgctttttt tagttggatt atatagatct

1201 ggaatccaac ttgaacattc gtaggatttt ctacccctac ttgtttcgct acttgtttga

1261 taggcctgca tcccacttca cattgtctac aaaaaatagg gattttcttt tccttaaaaa

1321 aaattctgtt ttcctttcct cttatctttt ttgtcctgtt gcggcacgag aaccttatgc

1381 cactatctag ctttgacatc aatttccact ttggatctgt aatcttgcat ctcgttgggg

1441 tttctgttgt tccttaggca tcgtgaagac tttttttgtt tctttatttt acttattttt

1501 atctattttc ttcaattggc cacccgtgag gtggaagtgg tttaggtcat tacgtcgttt

1561 atttatttat ttatatttta ctccccgaca ccgaaatgtc ggagttggtt aggccgccgt

1621 gacgcccgcc ggccaccgat ggctttacgg attcaaagaa tataataata gtttatttcc

1681 atattgtcct tccacttaac gtggacgcct tcattagttt ccgtaaatag tttccacgat

1741 caagggcctt ttcgtccgcc tggaaaaagt ctttcctccc tgtattacgg aaaggttcaa

1801 ctttgtttgc cctatttctg tggctgatcg tgatgttcct ggtccgggtg acatggacgg

1861 cccagatgca tgcatgtttc cgaggggagc catgtttggg taccgaatga agactttttg

1921 tgcagcgccg cggtgtgcat cgcctggtgg ccgtggcgac gcatccttta aatgcgggaa

1981 cgagggttgg tgtcgcgttg cacgacggct ggcttgttct ggatggacga gtgggccacg

2041 caatgaacgg tcgtatggtt atcgcttgct gcaaccgata ccgttactaa agggtccagg

2101 gtgcctacga aactatgttt gtatcctttt gttttccctt ttaaagagta ggctattttt

2161 tccctcaaaa agtgcccagt gtggacttcg ctttttggtg ttatgctcga gcactcatga

2221 gcgttccctt gtaagttatt gtcagtcata aaggtgcttc cagttaattt ttgggttttt

2281 ttgaatatag tgcttatttt tttccaaaca ataattcttt tggaagtgta acaaggtaga

2341 tgaagattat taaactcagt tatttccttt tttaggatat ccatgagaat aatttggatc

2401 actttttaaa aaaaatttat gagcatattt tccttttaaa gatagttttt atattttaag

2461 ttattatgtg cgacattatt ttagccggct agaatggaca tcattttcat gatctctact

2521 gggtatcgga gcaaggaaca aagtaaaaga cggcagatgc gctgtttccg cctcctgata

2581 caccagcctg tcacccacat ttgttattct ttttcttgat gaattggtct cagagaatga

2641 gcggctgtga gtggtgggtg tgtacggcca catgtgcccg cagttcaggg aataatttcc

2701 ccagcgcaag cccatccctt agctgccctc accatcggca tcgcctcctc acaagcgtcc

2761 acaacatcgc gtgctctttt ttttatatat aattttttta atgtatgata actaaataat

2821 gagttaatcc taaaaagggt gaaggaaagg aaaagccatc cttctttacc aattgctcca

2881 gcagtaagaa ccatcttttg tatgtgtcca gtcacctgct gtaaccttga ccgtctttgt

2941 taccgtctgt ggtggttgca gcgatatcaa taaagaggcg ctaatcaact aaaggcgatg

3001 g

>3 kb from ‘ATG’ of LOC105061227

1 tcggtaatcc ttcgctctca ccttcgatct tgactgtatg catgttctct tgtgatggca

61 catcatcttc cgatattctg ccagtccgta ttcaggtgca acatctagtc ggctccagac

121 tgactatccg atcggagaaa ggccacaata gatatctata tctttcttgg cgtagctcat

181 gtcacgtgtg gattcgagat tgtcaatttt gcataccaaa gatttacttt gccttcatcc

241 atagataatt caacattcat cgtttcgtgg cacaatattg ccattgctta atttatcaaa

301 tatcttatta attttaatct tcaactgttt acaaccagac aaaacctaag ccgtgggcga

361 tatgtcactt tgtgggcaat gtgtcaactt gcatcgcatg caatctgcga cactagatcg

421 gcgcatacaa agaaaagatc caagtccttt tataaacaaa gcgtaatatc agattattat

481 atgaatatcc gaatatttga aaagcatcag ttcagagtct gaattatttt aatctcctga

541 taataaaaaa aaaaaatcct gcagaggaca caaagggtca cgagcaaaga tattttattt

601 taccgcctcc tgactccaaa tgaccatcct aaccttacac cggatgcaca tcgggagggg

661 taaaatggtg atctggtatt ctgcggtgca gctcggccac aacgccgggt tgtcgccatc

721 aaatattaaa taaaaaaatg ttttataaaa cctcatttgg cgttcaaccg gggtacattc

781 aggcacaaaa gtccagatat ttttctgttg tgcgatcagc cgtccacttg ctttcccgtt

841 ctgagtcgag ggccacgaac ggcggagatg tagtcagtcg cagggccctc ctcagcgaaa

901 cgcgaccgag cgagagctta aataccgatc cgcctggttc tggagaagcc ctcagcggac

961 ggcgaagtgg ggaagcggaa gcgggagatt gatctctctc cccactctct ccgcgattaa

1021 tcgggcattt tccccttttc ttaggtacgg agttttttcc ccctcctcct cctttttcgg

1081 taagatttct gtattgtttt gcacggtttt tttgtttttc gatgaatgaa acgttcgtgt

1141 ggatcgaaaa cgatctccga ttgcgagatt tgttttttta gatcttccga ttgtcttcta

1201 tgatttgtcg acgatggaaa tgcggtttct ttttaatttt tcgtcggatt atatagatct

1261 aaatcgaact tcctcattcc agatttttct agagttacct gtttctctac tacccatctt

1321 acttgcattc tctatcaaaa atttcgaaaa atagtatttc atttttctta aagaatgttt

1381 cctctgacta tttttcaccc actatcaaat ctgggtggga cgaggaccgc atgccatcat

1441 cttagctccg ggatcaattt tcctctttgg atctgtagtc ttgcgtttcg taggaacttt

1501 gcttgtttgc ttatctggaa tcatgaagag tttttgtttc ttcattttat ttattgtctt

1561 ttattttgtt caaattgtta gatgcgcgag gtgcaagtgg ttttaaacat gatcacgccg

1621 ttgatttttc tatttattta tcctcttcca cgccaaaagt gttggagtag gtcagatcgc

1681 cgtgccgccc ggccgcggcc ggcggctcct cttctttttt ttttggattc caggagccaa

1741 ataacatctt tgttgttcca taactcctgc cggttcacgt ggatgccttc atgattgttt

1801 ctttggtaaa atggttacca cgatcggggg cctttccgtt cgtttgggaa aaaaaaaaag

1861 cctcatactt tgttctcgtg acacacaccg gcttttactg ataaacgtat ctggatcgtc

1921 cgatctgagg cgggtcgggg agtccctaga tccagtggcg tggacggtgc agatgcaagc

1981 gccgctcacg agggtggaat atttaggtgc ccaagaaaga cttcttgtgc agcgtgcgtc

2041 atggtggacg caccttgctc ggacgcgtgg acgcgggagt ggacggcgtc agctatcttg

2101 tgtttttatt ttattttcct ttttttagaa gagaacgcta cttttttttt ggtccgtcca

2161 taaaagaaaa cattcatttt ggacgttacc ggagcgttac acccgagttc ctgtgacgat

2221 tggtgtcggt ttccatcgtg tctcagtaat gtaaggggaa ttgttttctt agaaagtttt

2281 tttttcctta aaaacaacga gaaatataaa attttcttca agtttttttg taaaaagcaa

2341 agtattgtca cctgtcagcg attaaggtgc tcctgtcaat ttcagtctat ttcgagtata

2401 ctgcgtgagc caccgcacag ttttgtaaaa gaaatggaaa ttccattttg atgttgtgac

2461 gccatttttt ttggcttgta cgtagtctcc ttgcggtctg tgttagagga agtagcgact

2521 atgctattgg atcaatgggg tcagattacg caatctctcc ccttggtcat ccttcaccag

2581 acaaaggaac aaagtaaacg gcggcactgc catgtccgtc ctccgatgta ccagtttgtc

2641 acccatgctt gatattagat ctcttgttct ctcatactag aatagaattc actatttccg

2701 tctctaaaac ggtatcccat ccccctgcgg gcaaaccacc cctgtttagt gtcgtctggt

2761 caacatcacg tgctcacctc tccgtctctt tttaataatt tatttatata ataattactt

2821 ttaaaatgtg aaaaaaaaga aaagaaaagt tgttctgctt atttacaaat tggtccagtc

2881 gtcctgcagt accttgcgtt ctgtttgtat ctagtcacct gcggtaagcg tgaccgtttt

2941 gttgatggtg tctggcccgc agggtgctcg gtagagaggc cgtaatcgag caacgagaat

3001 g

>3 kb from ‘ATG’ of LOC105046119

1 atgaactgca ttgacaaaac cattactatt tgttataaag gttatagtgg ccatatttgc

61 tacattttcg tgatttcaaa tttttaatac caagtaatga ctttataata actgctacaa

121 tggtattata attcaagtca ttcaaagaag cagacacacc tgctctgaag cgaagaaaat

181 tatctttcag attgtagata gaggagatca aacaaaagaa gtaaacgtcg tcttagataa

241 ccgaaagtta tcggtaagtg gtaagccttc tgatggaagg gcactcggct ttctttttcc

301 atttgcttcc aatagttgga cctattcaaa aaaagtgtcg gccgttttcg tcaaagaaga

361 agggccttaa caggagcttc caattggccc catgccttac gctcttgtgg gtttgtttta

421 ctaaatattg aaaaaaaaat agacagtaac aaaaaaagaa ttgctttttt tctcaaatta

481 tgtttcacat acaaaaaaaa attacctttt gtaaaggtga gacaaaaaat ttggcatcaa

541 aaatttgaat tttgaaagaa gattatattg tattagggaa accatctaaa tgaaaaataa

601 tggccattat tttcattata ataatgtata ttatgataaa aatcaatagt gaccattatg

661 taaatgcatg ttgctagtat aacataaggg atcctccctc aaactatttt aattaatcac

721 cattatttta acataagagt ggaatttaga ccagttacag caagaaaata atgtgttttt

781 gctacacctt aatgtcactg atgatgcact tatgagatat gtgcaccata tgcaccatcc

841 ataacacaca tacagtatat aatgtaggat tctccagatg atgatgaaca aattgcagca

901 aacttaagcc atgtcctgac tcgacatctc aatttggaca ttatgggtga acatctcaaa

961 atatctgtgt cttctaatcc aaccgatcat ctaggtggag acaaaccaaa acctcagctc

1021 atcactttct caaaaacaga aaaaagaaag aaagaaaaac aactcgaccg aatcaattgt

1081 ctattttgtc attcctgcct ttattgctga agacaagagt cacgtaatga acgggcgttc

1141 gaagatgcgt taaatcaaag agggacaaag cactccaaag gaaattttgt gcatgccatc

1201 tgtatacgtt ttgaccctac aagacaaact tgaattttgt tcgttacata cttcatttta

1261 gctttttagc ggtcactttt catgtcattt catgcttatt ctcttttttc tttttctttt

1321 ttttttgata agaaaggaag ctacagccac aatagcatag cataaaactt aaagatagtg

1381 tttggttatt cttttcatat ttttgaaata gaaatataaa ttttcaaaaa ataactattc

1441 accgtatcta tttcagattt ctatttggtt agaaatatac tattccaccg aaaataagat

1501 gcaattaata cagtattatt ccaggtactt agaataagtt acaattaata ttcattaaaa

1561 atctagaata agctattcca tagtccatta tgaaatagct aaatcaagat agatgggaat

1621 aaagtgcaat taatgcagga ttgaccgaaa ttgctaaacc tacggagcct actgccactt

1681 ttttaatatt tggaggaata aaatgaaata aacccttact ccacttagaa taagaacatc

1741 caaacactac caaaagattc tacaaagtat ttgaacattt catgcttatt ctctttagaa

1801 aacagaaatt aacatgcttc gtgctcatcc ctttcacata tcctatatgt ttaagttcct

1861 tttactagac aatattgtat attatacatc atcttttatc gtgtagcttt aatttatacc

1921 ttttttgcaa ttataagctt tcaatttttt tgactgcaat atatgctttc aaatttatca

1981 cgaaaaattt tggcatgggt gataaaaatt aaatcttaag aacggcttat agcctcaaat

2041 ggggccatct ccatgatgat taataccatc acgcaattat tagaatacca catttttata

2101 gagaaaaaca aaaaagatta ggctaccaat ttcaatttct gacttacatc tattggttta

2161 tttaaatgtt tacttctgca tcagagatct tctatcaaat ttcagaagaa ttccgatgaa

2221 ggttaaaaat ctctaaagag atcttcatga agattaatgt attttttttc ttcatttttt

2281 gtttaataat attctatata ccaaccgtat tataattttt ttaaaatctt cttttctttc

2341 tcttgtaatt ttttttttta atctccaaat aaaaaccgaa tggctatttc tgcctccctt

2401 tcatgagaga gagagagaga gagagagaga gagagagagt actaccaac

2545 gagaga gagagagaga gagagagaga gagagagaga

2581 ttccaacaca gggcagctga gattgagcac aaggcgccgt ggaaaccacg agttccattg

2641 gcaacatggg aaacctggtg gccaagtgta gagctctctc acacaaaccc atgcggccaa

2701 cttgcagacc ctcgagtcat ttggactctt ccaagctcac cagccgtagg gttttttgac

2761 aagagggacc tccagtaaac gttaaacaaa ctcgcagctc ccacctttgg atccattcca

2821 tcgcttcaac ggtgggttag aagcctccgc gccaaatgca cgagtgctca acagcacgct

2881 cccctaattt ttctctctcc acctcctcac ttctctatat ataatcctct ctttggtgaa

2941 ccaccatcaa ccaaaccaac ggtatagtat acgtaggaaa taatcccttt ctagaacatg

>3 kb from ‘ATG’ of LOC105041997

1 ttttcagatc tctatcttat gttcatactt tttaggtgca caacatggtt ggatcaatga

61 taagaagcgt gtacccccac ctcaaaataa agcagcattc tattgcagct tagatgggaa

121 aatttgtcct acatcctcct aaatgacatg cacccacgtc accaccattt caactaaaga

181 caaggacatg acatgatgaa tgatcctatc tcaattatag ccaaccacta tgattctccc

241 atattttgac ttgcatgatc acaaaagagc atagtattgg atcggtatcg atgaaccaag

301 tcaatgttgt gctccaacca actccaacat tacacccata tatatatata tatacatcaa

361 tcctagaccc aatcaaattt caccttatgt acacgagcta gccaagctcg aaatcaattt

421 gatgtttgga gtttcaactt gttaattaga cctagcctaa gttctgagtt tgatacccca

481 tcaaacttcc aactatcaca atggcacatt tgtaaatgta ggcttgcata atgttattat

541 cgtagcgtta caatgatgcc aatagcacgt catcattcgc gagacgtacg tggcatcgtc

601 agatccttac acaactatag tttcgggggc accgaaacca agttggccca tttgtccaca

661 gccgacgggg gcctacgtac ccttgtctct ttgctcttcc ccctccttcc agattccatg

721 catgaacctc ctttttttgc tacgtccttt gccgacccca gtgcctttta ttttctcttc

781 aggtccttcc cttgggatat ccgaggtaaa tctccatatc caacagtaac ctccatctct

841 ctcttatttg ctcagtagta atcattttac tgtagatgga ggaagggtga gaaggttacg

901 tcgcgtttgt tctagtggag gatttaaata taataaggca ctactggcaa tgtacccgaa

961 accaataaga tcctatttgc atgattgcta tggagttctt tccggatgaa ggtgcaaggg

1021 gttgattatt aagcactccg aatgcgagtg tagggtattt tgttgtcatg tatgattttt

1081 gaaaaaaaaa agagaaaaat tatgatgcag catattgcta tggtgtttct agtggtacag

1141 atgatgggtc actgattcta ggtttataca tgcataaacc gaaaatgata aagaaatgtt

1201 gattttgcga caagcatcta gttggtgaga gttgctggca ttagcttgtt agttttggag

1261 tatttggcat aattagtagt gcaaagcttg gcctgtaatg ttctatgcca atcacatact

1321 atatcaatat cagttgaatt gtagataatg catggaaact agtccttttc agtgtgcgtt

1381 gttggttcct atgctatttg ttgcagcata gttggaaaca taaaagtttc atatttacaa

1441 cacttaatgt ttgcatagtg taggaaaagt tttctttgaa tctatttttc ttatcatcat

1501 agtaattttg aatgcgcttg catgagaata ataattggta tttacttcta gttatataaa

1561 gaaagaaata caaaacagaa aaataatgaa aatacaagtg acagcaaatg tttataactc

1621 cgggcacgat gcaacattta tatctaatat tttgttactc ctttgaatgt atggaacatg

1681 ccttcaccat catgtttaac atgatggcat attaactcca taacatattt tatcctatat

1741 atagctaagt tatttttcag caattatgtt atgtgctcat caattttcaa aaaaaaaaaa

1801 aaagacttga aattactata gcctaaagga tgctttatgc aaaacctact caacaatcaa

1861 ttgcaggtgt gactggccat tcattcaagt taggtgatat agttcaatga acaaaggaca

1921 tggtgatcta aaaatacatc tagtcctata ttttaggcct taaagtgacc catcctttgc

1981 aagttggatg aggcagatcc gcaccacatc accaattgct tagaagcaac aataatttta

2041 gggtccctaa atcatagcat ctatttaatg atgtcattcc caggagtcct agattttcta

2101 catccgagaa attaggtgaa aggacaaaac ctctaaaata ttaaaagaaa aagacagata

2161 cgatggtgcc cgcccgcatt ctagcccaac cactgacctt gatcctaacg aataggtatt

2221 catgtaattt taaacctgta agtagactac aacagtattt ttgctaacta gctccttctt

2281 accatccaaa taccatatcg tgcttatgtt tttcaataac caagctaagg aatttttttt

2341 ttatatgtat agatattacg aaaaaattaa ataatttttt tattaaccaa tttatcaata

2401 taaattaata atatttattt ataaaataaa aaaaatattt atttaataaa aaattaaatt

2461 attttttatc aatcattgaa acaaatatac attgttattc taaaaatatc ccatccatta

2521 cttccgatgt ccctaccatt caatcttcaa ccattcaatt atttattttt ttcaaattta

2581 aaaaaatata aaaaaatatt ataaaaattt taaataaatt ttactaattt attcaaaaaa

2641 ataataatat aaaataatat aaataataaa attttgaagt tatttttttc atgaaaaata

2701 tttatctata aaaatataaa ttttcagatt ttttttaaat atccataaat tttctcattt

2761 atttatgaga tttattcaaa aaaattcatt aaatacttca cattttctca cctaaataca

2821 agtccctatc cctccttccc ttctttgcaa aaccatcagc ataaaacccc cacccccaag

2881 cctttgcaag cccctcacac cagaagccgt ggcttctgaa aactctccaa aactttcctc

2941 tctctccaat tttcttctac tgcttccaga aagaaagaag ttttttagcg cgttgagatg

3001 t

>3 kb from ‘ATG’ of LOC105041997

[gap 1321 bp] Expand Ns

1322 tcttcctgc tctgttttct tttttttttt tgtttaatgg gctttagtgg gcttggtttg

1381 ggctataaca aaaatattag aaactctttc taatttttat tctattttaa tcaaagactt

1441 ctttaatcat catacgatta aagtatgtag gatgtgactt tctcttacta ggagtgatcg

1501 atttattatt gacctactca caactttcat acacaatcca ctatttccag aatatcttgt

1561 atataacttt agtcatgaat gagtatgaac caaaatatga attcacgtgc ataagatacc

1621 atggtggtct caggtctaaa gatcaggtac actgctctca cttagagaac catcttttaa

1681 catgtaagta agactccaca agatgttctt attgatgggt caattcagtg aattcattct

1741 ctaatgagca tccacatctt tgtatcagtg tctccataca agtgattgtg agatcaacca

1801 ccttctccat tgagtataca aaggatatgt cagttttttt gaaaatatta aacattgatc

1861 tccgactcaa tataccaaca attgaaaata ttttagatca gaatttttag attttaagtc

1921 tcactgacat gatcttatta tgattttaaa accattgtcc taatctatga ggctcatcat

1981 aatcttaata actataagat gcagtatatg atgaaacata aagtttttat ttattcataa

2041 aaatattatg tacatgagta gaaagatcaa aagaaatcct attgcaatat aaagtacgat

2101 tggcttgttg gacattattc tttcagacac aaatgtaagg atttaattgg aatcaaatgt

2161 atactttgtt tcaatgccct tccaattttt tttttcttta ggtaatatta aatttataag

2221 atctaaaaca cacatccaaa gattgaaatc ttttggtaat atgaaataca attgctagct

2281 aatttcaaaa ttgattattg attattctca tgatagatat atatgagata atttgatatg

2341 attgaaccta cacatcaaat ctttttttta tcacacctac gacttctttt tctttagctt

2401 tttcatcggc ctcccttcca aatggtatct tttctccctt cagcctattt aaaatgaaaa

2461 gtatatgtat ctaaaaactt catataatca aatatattgc catcaaattt tttttatctt

2521 ttttttttgt taacctatat gttcttttag ccttttgttt aattatttca accatttcat

2581 attttattat tagtaagtat attttatagt caattatatt tatctttttt atttttggaa

2641 aaattatact atgcatgcca gttttgctac catgcagtgt atatctgtat agacttcttc

2701 tgatacggta gattagagat tccgaggggg aatccatact tttcaccacc agtcagattt

2761 tccaggtcca accatcccat gcctcgacgg aattcgtttg gatgcatgga acggttgtcc

2821 aacctttgca cgctgttaac caaaaagggg aatggagcca ataaaagcca aaccaggaag

2881 gttcacgaat cggcgtcgtg cgtacaacgc acgtcaaccg aaagcggact cttttccgtt

2941 tcaacttcct cctaaaccca accaaatctc cctccttttt ctggctgaaa ccccaagcca

3001 t

>3 kb from ‘ATG’ of LOC105052365

1 atatattatt aatttaatat ataaaaatct aaaattatgt taatttttat tttagctata

61 ttttagattt aatttgatcg tgaactcaaa tttgagcatg actggatcga tgttctatcc

121 acgtcatcat ctacatacca tatcattacg aatcagctgt ataatcacct acctaagcaa

181 tttttgtcat ataatcatga ttattactag caaaaatttt tagtaagaaa aagtgatcga

241 aattaagaga aaaaacgaca ttaaaataat tgcaaaatag tagtgaccaa atttgagttt

301 ttttaacttt aggacttatt aaaaaactaa ctaaacttcg aagattaaaa atataatcaa

361 ttctagatat aatgctatca tatatactgt aaatagtgct cacttatcta atcattcact

421 tggaaacaaa tgtcattcag tgaaaaccta atatggtacc tgaggaaaat cttgaaatat

481 tatttgattg aataattatt tccattcgat gaaatcttgt taagtttttt aaatattgat

541 gagaactttg gctattataa atatgtcttg aggatagcta aaagttacat tgtattttga

601 gttgttcgta tctattgatt gataaatagc attgtggatt ggatgagttt atatattcgt

661 gatccaattt gaattaattt tatatctatt ttatctttat tttataaatt tattttttta

721 attttataaa atatatttat attagaatga tatgattttt tatcaaaaaa aaatctacta

781 atttacttct ctgaaaggac acaaattttg gagacttgtg gactaagaat tattataatt

841 tatgtttcta ttaaacgaag ccaatatttt atcaaaaaaa aaaacgaagc caatatcgga

901 agccgaggaa agcgaaaagg cacgcttttt ctcagggaaa gtggccaact gctgtctcct

961 aattctcgcc acccaccggc actgacacgt gtcgatgtga atactgtggg tcccaacttc

1021 cacgtggagt gaccggcaaa agcttcggaa gcgaaatttt tattggctgg agacacgtgc

1081 gatgcgggag aggatgatac ggacatggac ggccgggagg gagggttgac catggaggag

1141 gggagagggg cctttctgag tggggcccgc tgtgagggcg atacaaagcc cagggcaata

1201 accgggacgg tcgaatggga cggcttcccg gcggttgccg gtaagccgaa actggggctt

1261 tgctactgag ggcgctgata tatttgtggg attaccttcg gtccgtgctt ctgttttccc

1321 acctcaggag gctccgtcga cgtgtacaga ggtggcaacg gatagaattt agattaaata

1381 ttatattatt tatttttaaa tttaaattta atatttaata tttaaatttt atccaatatt

1441 cgatcagata agaaaagaga tatccatctt cgtatccaac ggattcgaag atactcatgg

1501 ataatccatt tctctatact caactcatat ccgctccata cctatctcat atctaaaaaa

1561 aataaacaac caaaataatt ttatacatat tatcaatcaa aataaaatta taaattcaat

1621 ataaaatata atttataagt ccaattttat aaaaatcaaa catacaaata aaattataat

1681 ttaatataga atatataaat aattaaaata attttatgca tattatcaat caaaatagaa

1741 atatcaaaat agaattataa atatatatag tcatgtatga attcggatat tatccatatc

1801 cataggttcg gatcggattt gagttcggat agaaaacagt actatccata tcttacccat

1861 atccgtacta tagttttcga attttatcca aacccagtca aatcttattt ttcgagtttg

1921 atcgaatttg gataggatgt atatctattg gatcggatcg attttgtcat ccctagacat

1981 gtatcggaga aaatgactac tgaacctccc aatgcaacca cctctctatt ctttcgtagg

2041 taatttttgg tcggcattag gtttggtatt tttctgagat cactgcacat tattctatcc

2101 taagtcaaaa aatatattaa atattgataa ttcatattta catctatatt tttaatagat

2161 ataaacataa atataaatat aaaataaata ttaaaattag tatctatatt taattttaat

2221 gatattgacc cgaagcagat atcagacttt tcatatttat tctgaataaa tataaatata

2281 aatataagtt tgatattagt tattaatcag attttttata taaaatttaa taataaaaat

2341 taatataaat aacaaatcat aaaagctgga aatgaaatct agcatggata attagttaag

2401 ggtctaaggt acaggtttta gactagcaca gtactaaccc gaggcaccgt cagtgtccaa

2461 atgggcaggg gcgttcttat aatatctcca acttttttct gttagattag attagaaaat

2521 gcaaagaaac ggcaaatggg catatgctct tgccagatgt gggacccaga tggccccgcc

2581 ttgccgcatc accgtccatt ccagacgatg acgtcatccc atgggatccg gaggcgccag

2641 ccaccaagcg ccggtggctc cgcttcctcc gggaacgctg acttggactc ccacctggca

2701 tctagtcccc ttctctcgca acattcacca aaatttaaac acccgcttcg cacccaataa

2761 tatatgttgg cccctttcgt cattggtccc actcgactgt gatagagttg cgttgggtgt

2821 atcgcaatgc ggtgtgaaga tctttccaag cttcgaccgg tatctcccgg tcaccctcgc

2881 gggtttcttt tacaattagc cctctcgctt tgctctctct ctccgcgcgt tctgagaata

2941 aggactcgaa atcctacaaa tccttggatt tgaggtgggg agatcgcggc ggcggggaga

3001 t

>3 kb from ‘ATG’ of LOC105050171

1 attcggccat ctggatgcaa atccggcccg aggtctataa aaatcgaccg acggtgcctg

61 tgatggggaa catcctcgac caaccgtttc tttcggacca tgtgatatct cttaagtaaa

121 tgacgtattt accctttacc ttaaacaaaa tatcgacgga attgccctgt aattaggggg

181 caatatgtga gggtaaattt gggagggaga caaggttttc cttcttgctg cagaaaagtg

241 gtgcagtctc acacgtgtgc tagcgacacg ggagctgcgg gggcacgtgc agctaccctc

301 gctcgcgcgc gcgctctccc tctcccaacg ggccctcctt ccgcgaggct tcgtaacggg

361 cgggatggag agaaaagaag gtgggagttg agtgttggac agctgtcaac gcgtaaccgt

421 tacatgcgaa aagtacgatt ccccaaatct cgcttctttt tgtttgtttt ctccggtcat

481 ctaccggtcc cctttctctc tcctgtcctt ttccgtttcc gtttcccttc cttctatttt

541 tatataattt tcttcaacaa cttaaaacaa agcggttcag aggcagtggc gcgcactcga

601 acgccatgac gtaatttgca cggatacacg tggggcaacc tcacaggctg ttcatccgta

661 cgctcagacc caccgagtag gggatcatgg aatggacggc tgcgatggcg gataaatcaa

721 atccggtcca gcaggtcaac acagtcaaac gaagacacta agcaaatgga tcagtagtta

781 agccgtttat ttcgcggaag gagcgttaat tcgttcgttg ggctcacatc ataagtagtg

841 ggcttttacg ttgggttagt ttttccgtag tgcgatagtg tcggaaatgt agtagttgag

901 aagccaacac gacgcatacg cacccgccac gctcgacaaa tacacgtata cgaaatcatt

961 aaataatgaa attaaaggag gaaaaaaaaa ggaatattta gcgttcgtgg gatgggagtc

1021 tgacctccaa tcaaaagccg cgggccaacc caacaacatg gcagtatggt acatcgtacg

1081 actaagagga aaagacgaga agatagagaa tacggaacag gggtagggat tatctattgg

1141 cattcaacgt gacggagacc gacttgctgc ttgttgtagg tgggaacgcc aaccggcaag

1201 ccatcccccc tgattcgtta acatatagaa cacaagatta agagtattta aagggggcag

1261 gggagggggg ccgggcaggc ggaaggagcg tgggctaggg taggggcata ccttcaataa

1321 acaataaata ttaaaaatat atccaaacac acatggccat cgatggcatt taattaatta

1381 acaataaata taagattaag aaaggataac ggaaggtcga tcgagagaga gagagagcgg

1441 tgtatcccaa accgagagtg ggagtccggt ttttggcagt tcgagtgacg gacagttgtc

1501 gatggaaaag gttagaggaa gggggggggg gnnnnnnnnn nnnnnnnnnn ncgagtagga

1561 aaggttagga ggaagggggg ggggggggag gaaggccgtt agggtgcggt tggggctcgg

1621 ttggcaggtt gacaggctgg atgacaggac ggcagtaatg atccgccctc tgattccgcg

1681 accgcgcttg tcatccactg ccacctcacc accttccatt gaaaccgttt ctttattgtc

1741 actctcattt tgaaatagag tttctagaat cccgtccatc aattatataa cgcagcatag

1801 aaacattata tataaattgc aactaaccat attatacaag atttaaaacc tccttagaaa

1861 tgctgtccag cctggcttgg tcaatactct catccggcta tctatgcttt gctcgcatct

1921 ttctcctctc taacccactt agccaccatc ttttatttat tattattagt gtccagagaa

1981 ttttatatta catagagatt atattaggta atagttcaat attataattc taccactagc

2041 cgtttccagt taagttttga atagccgaga tggatcgttg aatcgtacgt acgcccagtg

2101 cgcacacgta cggaggagca aggccagcct tatcgactgg acggccgaga ttaatcaagg

2161 gaaggatatg gacgccgagt cgcgtcaccc gcaaaatggt gacccgcatt catggcgggc

2221 ggtacgaatc ccccgcctga agcccagcag ggataagcta gtaacgttta ggtcaaaact

2281 aaaaacaagc cagccccaaa taaacgtgac gggctcggaa aatcttggac tccagttgaa

2341 ttgtcgaaac ctcaacactg ataccacctc agggggagac tatttactat tatggtctat

2401 ggtctgtgga gactttttcc agcttgcaga aacggatcca attccaggaa ctgtaacgga

2461 acaaacggag attgttagag aaacgcgatt cacaccaagc agtgacagta ggtggctagc

2521 tgccatccta ttccgtacgg gaaacgaacg cgttggggtg gcggtggagg aaagga

[gap 20 bp] Expand Ns

2597 gggn gggggggggg tggtgtgccc tattttaagg aaagtgaagg

2641 gcgggagaga gagaaaataa agacgggaag taatggagag agatcgcggc cgcgagcagg

2701 cgacgcgtgt acagatgcca gctgtatgca agcaaccatc tgcctggaag aagcgggtgc

2761 gccccctcgc tcgagtcgct atgaccggtc agctgtaccg gctctcccag tcacccacct

2821 cccctcgtcc ctcacttata tagcgctccc catgccactc ccatccccgt cctctttccg

2881 ttctctctct ttctcatgct ctctatagcc accgcctccc cttctcctcc ttagccaaaa

2941 ccgcaagggg ttcccttttt cctccatcga tctagagata gggtttgtta tatcgattga

3001 t

>3 kb from ‘ATG’ of LOC105044790

1 tcagaccgtt taaatcatgt tggtttggtt tggtttcaat tttttatttg tccgatttga

61 tttttaaaaa taccaaaccg tttaggagtg gttcggttta gatttgagta tgaaaccggt

121 ccaaatccga tcatgctccc cctacatgtt accattagaa tatcaactga agagttgagg

181 ttagtaaaca atcatagtga gatcgattaa gctttaagct atgaagttag ggtggtgaat

241 agagtgctga tgacactttg attttttttt actagcaata aaaaaggagc atcttgctac

301 cttcgatttc agtcaaaaaa aagaatagag tgctgatgat gattctactt gagcttgggt

361 tgagattcac ttgatgactc ttggtcaatc taaaatttga aaaggaagtt atatactaga

421 gtttgcctga cgaggagctc ttcgattgga ttccagaaaa ctaaaagatt tgagaaagag

481 atagaatttt tggagagaga aaaaagatag aatcatactt atatccactg actaacttat

541 ctatattctc atacttttta agatagataa gttactttct atagatagta tttactcata

601 aaatagaaag aaaatcttat ctatctagaa ggtggctaaa tcattttttc atcaaccaat

661 aaaattagta tttaatttta ttcctaaagt atcccatcaa tccttatttc tagtgcctcc

721 atcattcgac atttaataac tcagtcatcc atttcttcta gacctaaaag cataaagaat

781 atcatgagaa atataaaaaa attttagcaa cttacctacg aaagtaataa tacaaaagta

841 catcataaca aattttgaag ccacttttaa aatataaaaa atataaataa attatttttt

901 tatcaaaata ttgtttacaa aatatttata taaaaaatat atattgataa ataaattttt

961 tatccataaa aaaacggacc ttaaggatcc cttgtttatc cccttataca aagaaggatg

1021 gtcatcaagc ttggagagac atgccttaac ttctcattga tggttgccag gtcatgcaca

1081 tggctaagaa ttacaacttt gatagtgcag taacgatcaa ccaatcataa tggatcataa

1141 ccatcgataa aatcatgacc attgatacag atgaagattt ttgccttggt tgatctgagg

1201 gcttactttc aatgacatcg aagtgaggaa aacttcaatc ctaccaatcc atattgaagc

1261 caacccatag cacacttaga gtactcagaa atcattctgg gttagtacaa taggaaagat

1321 tgaccctcga ctctgcacaa tctttcaaat cccgcggtca tcgaaaatag ttaacgatga

1381 gcagataagg caaaagacaa cctctggatc atagacaatc tgatcaaaga tatcggagca

1441 ttgcacccaa tacgatgaag atgactccct atgttcccat ctcattgtag ctaccaacat

1501 ctatccataa ataccagctc ataagagacc ctaagatatg taatttctct ctcatactct

1561 tttctatctg ctcagagttc ctgtcttaaa cgatgtcgga actattttaa ctagagatat

1621 tttttgtagg ttcgaccatc gtcgtcttgc tgtcatcgcc taactccaat cgagtcaatc

1681 tggatcagga atttacagca acaatcacat tcttaatgga catcaacgga cttaattaaa

1741 ttgaaggcat tcttcgtttg ctcggttcta tgccccatgt caggtatttt ggattatacc

1801 aggatcagat tgacaagtaa cacgatgtca atatccctta tcatcatcat tatcatcacg

1861 gccatgcccc caaccgcgta gcgtcgcagc cacagccgca agaaagtcgt gacctcagaa

1921 aaaaggcgct gaggcagctc gcttgttttc tccgttaata ccgcgcgctg caccagcttg

1981 tgattgaaca gaataccggc gcagccatcg tgcgggcatt cttgatttct tgttgactga

2041 aagcccaggt tgatgcatcg gtggaaaaaa aatttagtat gcgacggcgg aaaagaaggg

2101 aaagtttttt ctatcttttt ttctaaaaaa tatttttaat caggtaaaga aaaggagaac

2161 agaaaaagaa aaaaaaagta aaaagagatt tgcaattttg tgagaccatc tagctggaaa

2221 actcccgtac cgaacttggt aatcatccca cgtcctaatt tcaacacgtg tcctccaaac

2281 ctcggaggtg gagagtaaca cgcgtcgcgc gatgaccggc

[gap 432 bp] Expand Ns

2753 ttcccctt

2761 aaactccctt cggaacatcg gatggaagaa tatcacttaa ttcccacctc cccatcatcc

2821 catctcgtcc catccctctc tctcttctct gtctcctttc cagttctttg cacgatagat

2881 aataagatag gccgtcggga ccaggggata tcgttacatc cgccgtaccc acgaagttga

2941 aaaggtagac catccatccg cccgaacacc cttctcgaca tcatctccgt catcaggatg

3001 g

>3 kb from ‘ATG’ of LOC105034225

1 ctcagcaaac cagcccaaag ccgtgcatga atcctttaac tcgccggagc atagcatccg

61 ggccgggggt ctttcggtcc acgcatatat atgggtatat gtggcgctga gggagcgcac

121 gtgcggagta cgttcacgag ctcgggtccg taccgcggcg cgtgtctccc aatctcccac

181 gtgtcgctta agcggccggc cgcggaatgc aactcatttc ccagaaccgg tttcggcccg

241 gtgggcccgc tcccaaggac aacgatggcg gtcgcgggct tgagagagag ggagactact

301 aaagtcacga gtgatataga gatggacagg agaagagcgg taagaagggc gggcattcct

361 ttcagacgga gagaactctg cctggagata tatataccaa ggtgaattcc tctctctctt

421 ctattttagg attgttcttt cgttatttta ctaatatttt cttttcagtg gatgggaccg

481 atgcatgtaa tctagatcta ctgctcttcg gatctcttct ttgttgtaga aatttggaga

541 atttatcaaa ggaagatttc ctctcaggta ctagatttga tttgccttct aatataatga

601 gtagaactcg tttctccttg tcctttttta tgtatctccg ctttatgctt tagagttctc

661 tggaattata tcgattccga tatttccttt gcctttttag cttattttct aagttttgtg

721 ctggtacttt tagaaattta ataagataga tttgaaaaat atgataaatt ctttcccatt

781 tggttgaatc ccgtttccat ggaattctct ggcgaatttt gttataagct cagctgagtt

841 tgtctgatta gattatgcat ttcttttgcg tgtctttgcg tccaaattgc agaattcatc

901 aaagtaattg aaggtagaat tgggaataaa attctaaata aatcctcaag ggcagtgttt

961 tcttgggaac caggagtggt ggtggccctc gaagtgttcc tagctgaaag aggaaagaat

1021 ctttcgtttt ttcctggtga cgcttctttc cctgatagga aactctggga tgtgggtcgt

1081 gtctaatcaa ccgtcctcaa gcagaagaaa ggaaaattag taaataacat ggacctgatg

1141 gagcctatcg tcgtatcctc cgtccttcaa agacgtggcc acattctccc tcctaagata

1201 tttctaatat ctttatatat ggtctgtagc atttcccatc ataaacgggt gtggatctta

1261 agatatttga tagggaaaca taatgtgaaa taggtgacag taacatatag aaaaattagt

1321 aattgtaaag caaaatatct aaaatgatag gatatcattt ctgcaatgtt tataagactt

1381 atgaattatt aatttttttt ctatatttga gttattaatt gatttgtgaa aaatttaagt

1441 gttttgagat cagtccagaa catgactggc agtctgttgg cactccatgg tgaagttatg

1501 tacttttgtc catatgattc ttggtttttg tctcatgcat tgtgtagtca aaaggtggat

1561 agcgaaggcc ggatatttcg gtttctggaa agcgtaatac gcagtccttt ccacaatagt

1621 ttgattatta ataattaatt ttcagttctg catgtatcac agaaatttca tataattatg

1681 ttgtccattg aatcatagta ccatgaggaa tcaaaacaac aaaaaaaaca aaacctttga

1741 gatttggaat ggaggctatt catttacagt tcaattgcga taagagtgat gctaaaagta

1801 cataagaagt tggcaaataa tactgaaaat gagatcagcc agcctaattt ttcatcctcc

1861 aagtaatatt atggtactat tttcaagatc aagaaaagca aaaaaaacaa aaaaaaaagg

1921 acctttgcga atattttcgt gaattgataa tcttatttcc agctacttgt gcaaaatttt

1981 tagatcatgt ttgattagga tatattatat tatgagataa tatgataatt tttaaaaatt

2041 atttatttaa aaataattat gagaaaaaaa aattattata ttttattggt ggaaatatta

2101 gtccgaacaa tgacatcaaa aaaaaaaaaa aaatctgtta aagataattt tgtataaaaa

2161 atattagctt ggaacaaaat taaatgacgt gcggttgggt gggggttgaa tctgagctct

2221 gttcccattt caggaaagtt tatcctatgc tgttccgcac gaacattttg aaccaaaccg

2281 agtcctgcgt ccggagggcc aggtaacatt tagctccacg ggttgcaccc cacggcgcat

2341 caagtgaaac ctcagcacta ttcatatatt tcttcaagaa gcccacctcc tcctccatca

2401 atgagaacgt accaaacttt gtgtacttgt taattaccgg gttaggcacc tatttttggt

2461 agcaattttc taatcagtac tcgtacgatt atcaccaatc ggtcagtttt ggtccaacga

2521 agaattgcgg caccatcgcg ccgtacattt atgctacgat cttccctgcg ttttattctt

2581 gttttcttgc gggaattgta gaatcgccac tcgtgactcc taattccaac gttcttttca

2641 ttggcagtgt agtacgaata ggctcttggg acaagacccg taaatttgtc cgaatattga

2701 ggctggaaag cgtacagagc ggaaaagaga gggagagaga aggtattcga cgcaggtcat

2761 cctcatctct ttccttggaa atcgtcgccg tcgcttctcg gtgcttctgc aacgactccg

2821 tccgtttctt ccttcttttc ttcacctctt tttcttttcc agtgtaatga aacgaacttt

2881 tccttttttg tcgttttgca ccccctcctc tcgctagcta gctctgttta ttcttctcta

2941 aagtggaagc agttgttgag cagaggagag cagaagggca agaggattaa ttaatcgatg

3001 g

>3 kb from ‘ATG’ of LOC105048209

1 acttaattta tgagtgagaa gtcatacttt atcatcaacc atgtcacttt tttgatagga

61 acatttcttt gtgatcatgc gtgacggccg tgtttgtgtg aaggagagat gatatatttt

121 tttttgatct agtgcgttac atctacatat atctattttt ttaaaaagaa aaaatcttgt

181 tgacctaaca caacttgata aaaaaatatt tttatagtag gttttcatgg aatggctttt

241 gtagttgcgc tacctataga agcttatctt ccatagagaa gtttgttatg gtgttggaaa

301 ctataacttt tggtcattgg attctccctc gaacaataaa tatgaaagga aattgtctcc

361 ttgattcatt tcatttgcat ttttaacata gtttttattt ggtggtgtgt gtctctaaaa

421 gtaattaaat ttgttatgga gttaaatata tatatggtgg attttgagat tcatgtataa

481 tttcatgcac aactcttaaa atattctgta tgctttagtg agggcctatg gagacctaac

541 cctctggaga tggagtgtgt gtgtttttag atacttttca atttattaga tgtatgcgcg

601 cgcgtgtgtg tacacttttc aatttattaa acggatagca tccctatcca tctagtaaaa

661 tgactaaata aaagaaatta aacttaacgg aaaatacatt tgtgaaatta taatataatt

721 taaaatattt ttctataata taattttaaa tatatttttt tgaagaaatc atatacatcc

781 tcttccaatc ggtcaattga tgatgtatat cttttttttg gtggggggag taaaaaattg

841 acgattgtat atcaaatatt ctttcagcat gcaatactag catataaaga tcagctagct

901 gtattttgat gaataaagca gtgacgtgct tttgattaat tgaatttaaa tcctttctag

961 tttagcattg aattgggaaa attcagtccc gctgtcatga ccatctttct attgcgtaac

1021 agtaacagtt gtaaattggt gaaacgcata caatttatga aaaagtatcg tatcgtatcg

1081 tatattatat tatataggat ccaatcaatt ataatcttta tgcaaaggat aaatggctgc

1141 aactgaaagc gagggacagg aacgcattgc gagaagaacg ggacggtggg cacgcccatc

1201 tcgtgctgta aaaggacgaa cgaccgccca tcgggacaac agccccgtcc gagctccgta

1261 aagttggtcg gaccacccta ttggattcgt tctggacgag gcactccttc tatcttttgc

1321 cttaaaatac ctacggtggc acgtgggatc gtggatccca atttcccttc tatcaaccgt

1381 tcgaatcaac aaatggagcc ttttctgcta tcagttcgac agacggtcgg ttagctactc

1441 aacacgagca ttgtctccat cttctatcat ctacactacc tgagcggaca gtgatgctaa

1501 ataagtctct ctctctctct ctctgggtcg gggtaaccat aacattagga agaaataatt

1561 aaaaacaaag tatatcaaaa aaaatgcaac agtataatta gtgcatttct ttcaataaaa

1621 ttacatgcat gccaccacta gcaccgggta tccggaaata gcctcgagat atccgggaca

1681 tgcatccact aaatggtgga tattcaaata tagggtgaca atttttcaca gtacattgag

1741 cgttcgattt gatccgattt aaataggata ggttttatct gaactccatc aaatttagag

1801 gtagatatgg ataatagtaa aatctatcga aaaatcgatc tgatatatat ataattttta

1861 atgaattacc tctttgagat aaaaatatta atttatatgt aaacaatcaa attttatagt

1921 tttatctaat ttttcttgtt aaactttatc tatcccgtcg cttgttgaga tgagaataag

1981 gaaaatatgg acaataccct atttgatcca tactcaactc ttgttatcca cgttcgtatg

2041 gaaattgtgg caactttcac tttcacgtat ttgttatggg ttgattgtct caagattaaa

2101 ggtgatgtga acgaagatat caataacatc atatttagtt tcgtcgtaat gatccgatat

2161 agcagtaatt ctaaatcatt cttacttctt aaaacattat gatatttttg atgtcggata

2221 tctaagatca tcccagaacg atgatcttga gttgaaattt gaagataaaa atacttttca

2281 atcgaataaa aaaattaata tatcaatata ttattaatat attatatcga tatgttttgt

2341 tatattaata ttatatatca tatttatcat atcatatttt ttattcaatt ataattttaa

2401 attttataaa attaataatt tatataatta ataaaattat tttaataaaa tatattaaaa

2461 ataatttcaa tattacatga tcactgatct aaaatcaacc tggtgatact atttaggtca

2521 ccaaaggcca ctacggtgta cccagaaggc caacatggtg tgctgcaagc ggccagggga

2581 aaaaaagctc accgcgagtg catacattct tgcggcagca gagagtggca tatcgataga

2641 taagccgggg aagcgagggc aaagccacta taaatcgggt gaggataaga cggcgttcgc

2701 aactggtggg gcacccgcga tttttatatc gtttcgggtg gggggtgcgg acggtggagg

2761 cgaaagcaag gcgatcagag acaggctccg agccgaggct cggaccatcg cggtacctct

2821 cttctctttc tttctttctt cgaagcgaat cgcaggagtg agagagatag ggataagaga

2881 gagagaagag gagaagcttc gtcgcgaggg aacgagggcg ggcatcgggg gagagagcgg

2941 cctttcttct cctgttagct tggtgggggg tggtggtgtc ggggtcctta aagctggatg

3001 g

>3 kb from ‘ATG’ of LOC105048609

1 aagtatttaa acccctctct ccatccccct tcaacattaa gataggtatt atttatagat

61 tgtggattga gtatccaact ctatgaaaat aataatatgg ttcaaataaa ataaacattt

121 tggataagat tgacctaaaa tttttttatt tgattactac ttctactctt tatgttgagt

181 ttctaaaatg cttgttttac tagtaatacc caaatttgaa ttctagatct tctccaaaga

241 caagtcagag gggtggcggt ccaattagct aatagccatt ggcaaatata accattttgt

301 atcaatcaaa attttcctct atatcgatca atatttcaaa gtctaagcaa gaaagtgtca

361 tatatatctg aaccataatc tctatgtgaa tgtaggaagc tccacatgcc attcgccacc

421 agcttgcttt ctttgttaat atcacttgtt gcattattct cttcacatga actaatgcct

481 agatagttca ataaacatgg ccttttatat tctaattatc tataaattta ctttacatat

541 tccttgcacc ttttgtcctc gcgtgagctg gatcaaaatt agatgcatgc attttttcca

601 ttcttcctct tgctcttgtc ttgatctcat agaataatga aatgtgaaag aagataatgt

661 cctggtcccc tactggtgat gatcatatca atccatcaca atttctagtt ggccatcctc

721 tgtccatggc tgctttttat aataccttgc ctcagaaaga agccataagg cttctaatac

781 attataaacc tggtagatac gccatccata tcgtgaagtc gtgatccata aataaatcta

841 gcccaccttc atcgattaaa aaaaaaaaaa gagttagtgg atctactacc cttcacctat

901 cagtatttgt atatattaac ccttgtgatg ctatccattt aatacaagga tttttattat

961 gaatacgcat ccactaaatg aattcaccaa taagttccaa ctaataaaag aaaattatta

1021 cctctgtacc atgaaaagaa aaaaaaatta tgacctggat ttcccaatgc cacccaccat

1081 ccccttacct ttattaccgg ccatggcatg tgctagttgg aaaaattctt tgtataccat

1141 gagcggtgta aaaaattcga tatagggcat attattttat atgattgatc catgtagcca

1201 ctacttttca aaatgcattt aatgcttgta gttctatttt ttcgtttgaa aattttgaat

1261 aatgaaaata catcgattct ttgaaaaaga ttatgacatc ttatgtccat attatgacat

1321 actgtataca aaaagtcata attttaacgc aggatgttat aatataccat aggatatcat

1381 aatttttcaa agagtaggga tatttttgtc attcaaaatt tttaaacgaa aaatcgaaac

1441 tgcaggtatt aaaagcgcat tgaaaaatag ttgaacaaaa atgtcctttc tatattatga

1501 catcctgagc catattataa cgttctgcat gcaggaagtg ataatttgac gcaagatgtc

1561 ataatatgca caatatgtta taatttctga gctatatttg atattctgta tgtagaaagt

1621 cataatatgc cacaagatat cataattttt ttttaaaaat aggaatattt tcgtcatata

1681 aaatttttaa ataaaaaaat aaaactatag atattaaata tacgctgaaa gatgataaat

1741 atgtagatca attatataaa atgatgtatt ttatatcaaa ttttctatac cgtcagtggt

1801 gcacaaagaa tttctcgcac tagttgacct ttatcattta gctcttacca ttattactgg

1861 tccattgcat atgagagttt tttctacgtt ggtgcaccta ggatataata tttatttgat

1921 aatccaatcc aatccaatcc atgtgccttg cattagtaca agcatttgct gttccctcaa

1981 taattgctcc gcgggactca tatgtattgc ttcatggaca tcaaggtctc aacaaatgac

2041 agttctcctt aaatactgtt ttttgttata atgagaatac tacctgctgt tcatgcttgt

2101 tggaatttgg cattggaggt taaactggtc ccctcttcgt ttgtcattat ttgtgttgaa

2161 gagtaatagt ttcatattgg atagatcaaa aagtcttata ctatttatat aatcttttgt

2221 accatatcat cgtcattatt gagatagcta taaccgtaga gagcaataat tgagcaaatt

2281 cgatacagta tgttatccta tgtgcctaat aagatccgct taatcatagt tttggatgcc

2341 aattcaactt gaagtcgaga gatctcatac ttatcaattt cttaaattaa atataataat

2401 aataataata ataataataa tattattatt attattatta ttattattat taattaa

[gap 20 bp] Expand Ns

2478 att aaattaataa taataataat aataataata ataatattat

2521 tattattatt attattatta ttattattaa agttaaacga atgatatctt ttccctcttg

2581 aggctaaatc tttatgttta aattagaata gttagaggta aacagatgac gtaaaataac

2641 aattattaaa ccttcgtgag aacatggcgg acaaaattat gccccataaa aaacgctaca

2701 tgggtctaga tcttggagac ccctacaaga gaatttggat gcgcccaccg ggaagctcca

2761 ccacgactac ggtatcggtc tgtggctgtg tacaatctcg cacgtgtcaa gctcccactt

2821 tcgtcctgtt acgaggaatc gcgtttcccc ggctcgggtc ccccacctct ccctcacctt

2881 tatcctctcc ctccccttcc cccgcctccg ataccgaaca aaccgaacat cggctattct

2941 tgctgcctga tgtgagccgt agctggccat ccgcggccgc cgcagcctcc gtcgttaatg

3001 g

>3 kb from ‘ATG’ of LOC105058890

1 atttggaaag gttgatagaa aaccccatac aatcccgtca attcatgcca ctactgagag

61 cagctagcta tcgaccagat aagacagcta caactattca agattgagaa attaggtcga

121 agatcttggg atagcacatc caaaagagtt ggaacagtct ccaattcttc tctctcacag

181 ttctcccctc tataaacaga gataatcaga ggactctcaa ggtatacaat tctcttgcat

241 actccttcct atctgcccag tcggtttctg atttgagcgt cggagagtcc ccaccggagc

301 tacctctagt cagaggctta ttttgtagat ctgatcacca tttcctcact cgttaccatc

361 tcactccagc cagttcaacc caagctagga atctgccaca acaacaacaa aacgagaaca

421 tgtttcttac tccttcaagg aatagttaaa atcactaata acatttgtca tttcaaataa

481 attggcaaat ggctcgagtt gtatctttca tatgaaagaa ttattgttct tctttctcag

541 cttcaatcgt tggggttgtg gtttacacat ctttcaaaat atgactaatc ttttccattc

601 atccgcaata caaatcatga aactcatatc atattttgaa agatgtgtaa agtacgatct

661 aatattgaga tcacaaaaaa atattgatca ttcttttatg cgaaagatac aatttgaggg

721 gatgattttt ttttttttgt cttagttagc tctctcttgt aaccaagatg gaactcacca

781 atctgagggt taaaactgta aatgagattt gagcatggtc tcctgcctat tggactcgaa

841 aataggcaaa gcatgtgcaa cctgatgctt tccttgcact aagcaatagc ctaattgcag

901 tatcaagcgc gatatgacga ataacaccta acccaatcta aaccggctgg accattaatc

961 taacctggta ttatattggg tccatgttat tggcaccaaa agcaagcggc attcgcggtc

1021 ccatctcaga gaatctcgtt tgtccatttc gctttgagcg aaagccggcc aatattcggc

1081 ataaagaaat ggacggacca aaacactgtc caatggtgag gataccggag tctggatctc

1141 ctgtgatgct acttttcgca cagaatgtga caccatggta aataccaacc atgtcatcta

1201 tttgggaaac atgcgggttt taatcgtgcg acttttgtac gatagtatcg taaataatta

1261 ccatatcatc gattttaaat ataaataatt tatttatcta atttaaaaaa taaatagaag

1321 gaggatgtgt cagctgtgat attgaagcca tccatttcca ggagtaatga tgtggcaacc

1381 atccgataag gatacaatgg tccgcggtgt aaaagtcgca ctgcggtgga tcccgtttcg

1441 ggagatgcac gcctgcataa aagggaaggt tctctcttat tcccaccgat aggtgatcag

1501 atctcgttct cagtctacac tccatcggca tcgatctcgt ccagatctct ctccttctta

1561 cctccgcctt tcttcgtcgg ctcctctttt tacctctttc cgtcgtcgat tccggcgatc

1621 gttggtaaat ctgttgaggt acgttctcgt ttgtacgcca ctgtctgctt ctcctcgtgc

1681 tcttagtttc gatcggaaac cctaattgga aacatggatc tctttgtcat ttccttgggg

1741 tttgatctgt ggaaagaatg ggaaatgaaa tgaaacggtg gaaagctttt cctttttatt

1801 agaaaactgg gaagaaataa tataattgag gtgatcaata ccggtttctt gctggagttt

1861 tggtgatttt gtgtaaccat gctggggtat tcaatttttt ttgaaaaaaa agaagttttt

1921 tcgttctttt tgactccctt ttccaattta tctgcgcaat aagctggcaa tatgttgttt

1981 ttttttttgt ttaggtgctt ttggttgatt tcttcgtgaa tggattgtcg cctgaatctg

2041 gtcttttcgt gggttctgat atttgaataa ttagaattgg aactaattgt ggtggtgaat

2101 tagatgggga acaaatggtt acttttgttc acaaccaaaa gaccagattc aggcgagaat

2161 ccatccacat gttctgtgtt caaccaaaac acgggattca gatgagaatc cattcacatg

2221 ttttgtgttg tggttactgg tcggcgtttg gttatgttct gtgttgtggt tatgatacct

2281 tggcagaaat tacatctggt ttcaaaatta gcttttgaac agtttctgtg cttatattgc

2341 cgtgatctag ggtttgcaaa tttgtattta tttatttatt taaacattta aaaaatgatt

2401 atctgacttg catgctgcaa agtttggagt agatcttcat atttctcttt taaccatctg

2461 ctcggtaatg catcaaactc gagtttttgg cacatcctaa ggttcatgga actgacagga

2521 cgtgatatca tgtctgcatc tcggtaattg taaaataaat tcattgagaa ctcaagatta

2581 acaatccaag gcatattagg gtggtgggat atacggattc aggtcctaga aacgtctcga

2641 gggacaccga gatggggtat catctcatgt gttgagacag actgtcccgc tagtgtccta

2701 gcatcccaat cggaatgcct cgggacatcc tctatcccaa gtgtcgggac agggcaggac

2761 ggcgatgcat cttattccat aggaaaattg ggacagtctt gtccaatggg atttaaaatc

2821 ttggtgggat ataggaaaaa agctgtcaaa gcttttttgt ccattgagaa tcttcaaaga

2881 gccattctct caaatctcat tttactttta tttgcaactg cagatgccaa catccatgtt

2941 tttttttgtc agttcatgga ctttgaattt ctcataccgt ctagtgttac agtgataatg

3001 g
