# Supplementary material for: Abscisic Acid Improves Linoleic Acid Accumulation Possibly by Promoting Expression of EgFAD2 and Other Fatty Acid Biosynthesis Genes in Oil Palm Mesocarp
Source: Front Plant Sci. 2021 Dec 3;12:748130. doi: 10.3389/fpls.2021.748130 (PMC8678531; doi:10.3389/fpls.2021.748130)

Supplementary figure 1 Differentially expressed genes of CK-3 vs. A2-3. **(A)** Gene ontology (GO) annotation of DEGs in CK-3 vs. A2-3. (**B**) Top 20 term of GO enrichment of DEGs in CK-3 vs. A2-3. (**C**) Kyoto Encyclopedia of Genes and Genomes (KEGG) annotation of DEGs in CK-3 vs. A2-3. (**D**) Top 20 term of KEGG enrichment of DEGs in CK-3 vs. A2-3. The size of the bubble indicates the number of genes, the blue depth represents the size of the Q-value.

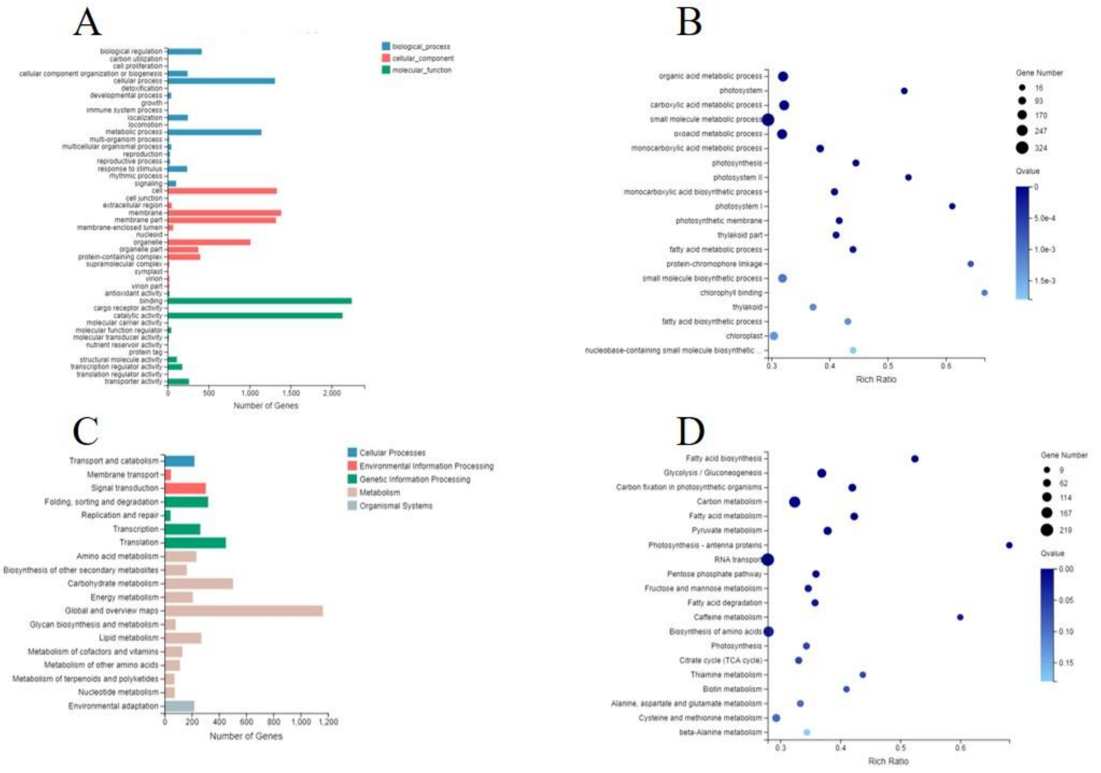

Supplement: Supplementary file 2 [file Image_1.pdf]
