# Supplementary figures and images for: Abscisic Acid Improves Linoleic Acid Accumulation Possibly by Promoting Expression of EgFAD2 and Other Fatty Acid Biosynthesis Genes in Oil Palm Mesocarp
Source: Front Plant Sci. 2021 Dec 3;12:748130. doi: 10.3389/fpls.2021.748130 (PMC8678531; doi:10.3389/fpls.2021.748130)

Supplementary figure 3 Phylogenetic tree analysis of FAD2 in different species.

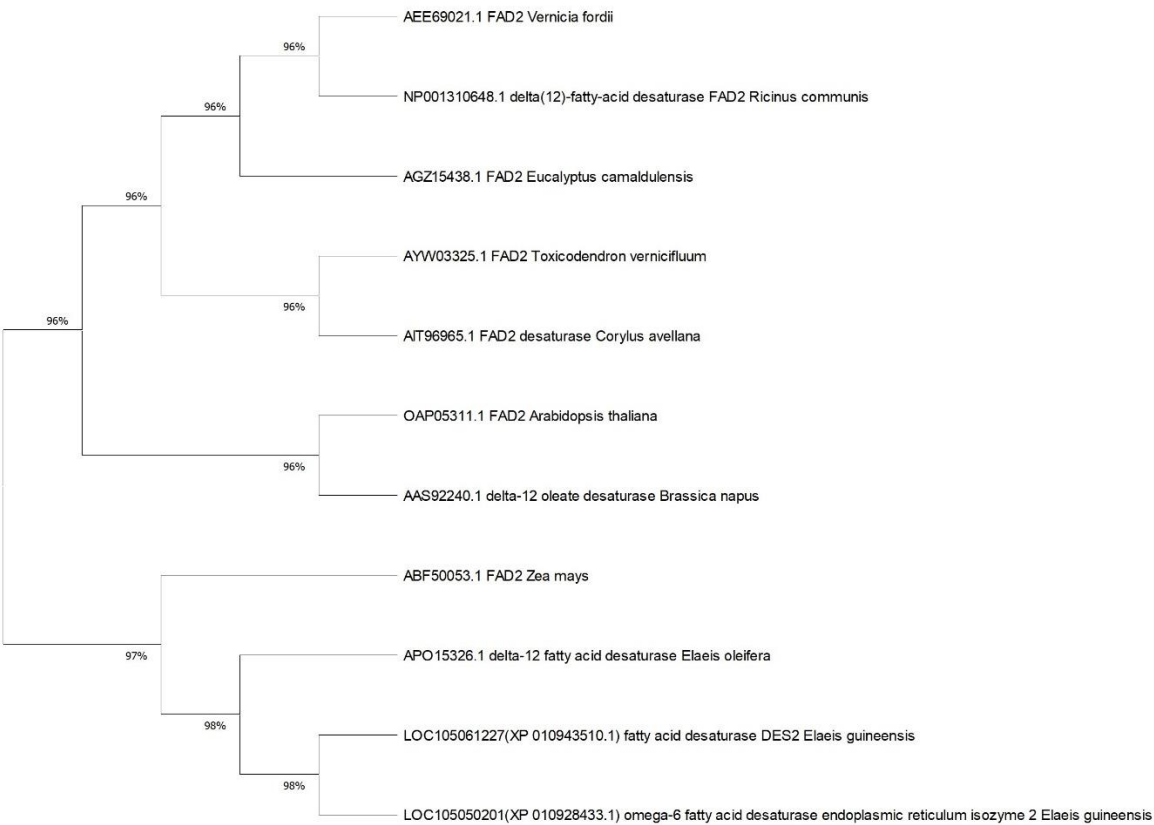

Supplement: Supplementary file 4 [file Image_3.pdf]
