# Supplementary material for: Abscisic Acid Improves Linoleic Acid Accumulation Possibly by Promoting Expression of EgFAD2 and Other Fatty Acid Biosynthesis Genes in Oil Palm Mesocarp
Source: Front Plant Sci. 2021 Dec 3;12:748130. doi: 10.3389/fpls.2021.748130 (PMC8678531; doi:10.3389/fpls.2021.748130)

Supplementary figure 4 Co-expression network between CK and A2 during mesocarp development. (**A**) Module test result by WGCNA. (**B**) Network constructing by 1348 genes. (**C**) Enlarged view of big network. (**D**) Enlarged view of small network. Orange circles represent genes involved fatty acid biosynthesis and ABA response.

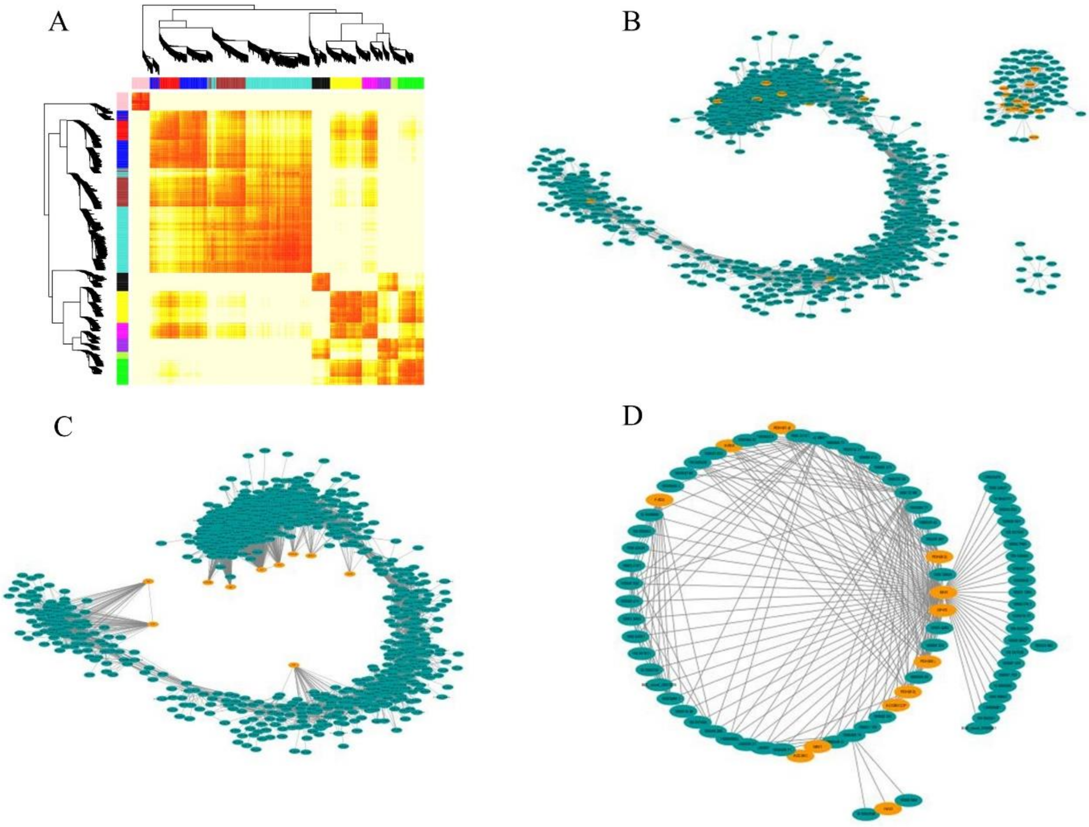

Supplement: Supplementary file 5 [file Image_4.pdf]
