# Supplementary material for: Abscisic Acid Improves Linoleic Acid Accumulation Possibly by Promoting Expression of EgFAD2 and Other Fatty Acid Biosynthesis Genes in Oil Palm Mesocarp
Source: Front Plant Sci. 2021 Dec 3;12:748130. doi: 10.3389/fpls.2021.748130 (PMC8678531; doi:10.3389/fpls.2021.748130)
Supplement: Supplementary file 6 [file Table_1.docx]

Supplementary table 1 Fatty acid composition in mesocarp after different treatment (mean±SD, n=3, μg/g)

| treatment | CK_5 | A1_5 | A2_5 | A3_5 | A4_5 |
| --- | --- | --- | --- | --- | --- |
| C10:0 | 8.63±2.54a | 6.20±0.54a | 7.54±0.25a | 6.56±0.95a | 6.97±0.10a |
| C12:0 | 45.96±6.90a | 28.28±0.89d | 45.08±2.47ab | 39.26±1.91bc | 37.80±0.53c |
| C14:0 | 1240.50±100.53b | 782.36±6.95d | 1439.02±11.75a | 895.33±2.75c | 949.23±1.70c |
| C15:0 | 47.39±5.31ab | 41.82±2.18bc | 53.00±3.60a | 40.66±0.50c | 42.50±1.22bc |
| C15:1 |  |  |  |  | 11.61±0.00 |
| C16:0 | 92929.54±14219.78a | 72071.46±1825.09b | 94289.18±1736.70a | 69950.95±1403.92b | 80854.15±2537.13b |
| C16:1T | 8.26±0.00 |  |  | 6.03±0.00 | 12.24±7.56 |
| C16:1 | 140.65±33.70a | 100.40±8.32b | 137.94±13.77a | 120.76±5.50ab | 138.84±8.44a |
| C17:0 | 166.61±21.50a | 155.87±5.36a | 159.91±5.83a | 159.54±4.90a | 163.70±5.45a |
| C18:0 | 9787.29±870.95b | 8190.28±43.69c | 11025.28±136.10a | 7919.01±344.15c | 8686.50±177.27c |
| C18:1N7T | 23.63±0.00 | |  | 25.49±0.00 | 16.90±0.87 |
| C18:1N12 | 20879.38±9533.69a | 33077.79±5247.84a | 31520.41±7897.64a | 32454.61±1215.61a | 31785.80±7794.40a |
| C18:1N9C | 104320.67±15054.26a | 89962.36±1065.25bc | 99429.10±1311.11abc | 87893.34±2709.64c | 100714.89±2153.19ab |
| C18:1N7 | 1249.08±135.63a | 1026.97±14.80b | 1199.20±28.34a | 973.41±7.82b | 1262.43±2.28a |
| C19:1N9T | 202.52±111.46b | 349.12±90.08a | 403.99±146.13a | 330.81±7.69a | 330.12±82.92a |
| C18:2N6 | 14437.77±1165.78b | 10950.83±47.89cd | 15640.40±279.58a | 10597.19±557.82d | 11902.61±226.21c |
| C20:0 | 818.30±81.26b | 624.71±6.26d | 964.89±14.87a | 613.15±13.44d | 731.52±10.06c |
| C18:3N6 | 15.61±0.00 | |  |  |  |
| C20:1 | 339.15±39.31a | 233.80±7.53c | 354.41±5.64a | 225.14±10.61c | 280.71±4.46b |
| C18:3N3 | 746.10±84.57a | 539.61±0.60b | 760.14±9.45a | 485.46±15.78b | 553.83±19.18b |
| C21:0 | 11.11±0.17 | |  |  |  |
| C22:0 | 93.59±24.54a | 34.41±3.03b | 58.86±17.21b | 32.41±9.00b | 45.63±11.59b |
| C20:3N3 | 16.47±5.08a | 30.24±7.05a | 28.48±7.67a | 26.98±0.00a | 17.16±0.00a |
| C24:0 | 118.23±23.36a | 45.80±5.80c | 79.95±22.74b | 43.69±6.57c | 69.41±15.03bc |
| oil content | 247611.07±25597.82a | 218250.25±3267.69bc | 257596.76±10643.91a | 212800.78±5033.69c | 238583.34±3117.48ab |

Notes: CK_5: 24 WAP with 0 μM ABA; A1_5: 24 WAP with 10 μM ABA; A2_5: 24 WAP with 20 μM ABA; A3_5: 24 WAP with 50 μM ABA; A4_5: 24 WAP with 200 μM ABA; C10:0, Caprate; C12:0, Laurate; C14:0, Myristate; C15:0, Pentadecanoate; C15:1, 10-Pentadecenoate; C16:0, Palmitate; C16:1T, Palmitelaidate; C16:1, Palmitoleate; C17:0, Heptadecanoate; C18:0, Stearate; C18:1N7T, Transvaccenate; C18:1N12, Petroselinate; C18:1N9C, Oleate; C18:1N7, Vaccenate; C19:1N9T, 10-Transnonadecenoate; C18:2N6, Linoleate; C20:0, Arachidate; C18:3N6, Gamma Linolenate; C20:1, 11-Eicosenoate; C18:3N3, Alpha Linolenate; C21:0, Heneicosanoate; C22:0, Behenate; C20:3N3, 11-14-17 Eicosatrienoate; C24:0, Lignocerate. Different letters within a row indicate significant differences between the means (p < 0.05).
