# Supplementary material for: Abscisic Acid Improves Linoleic Acid Accumulation Possibly by Promoting Expression of EgFAD2 and Other Fatty Acid Biosynthesis Genes in Oil Palm Mesocarp
Source: Front Plant Sci. 2021 Dec 3;12:748130. doi: 10.3389/fpls.2021.748130 (PMC8678531; doi:10.3389/fpls.2021.748130)
Supplement: Supplementary file 15 [file Table_10.docx]

Supplementary table 10 ABRE cis-acting element prediction of genes involved in ABA signal transduction and TFs

| Gene name | Gene id | sequence | position | Matrix score | strand | organism |
| --- | --- | --- | --- | --- | --- | --- |
| PYR | LOC105048209 | ACGTG | 924 | 5 | + | Arabidopsis thaliana |
|  |  | CACGTG | 1340 | 6 | + | Arabidopsis thaliana |
|  |  | ACGTG | 1341 | 5 | + | Arabidopsis thaliana |
|  |  | ACGTG | 2029 | 5 | - | Arabidopsis thaliana |
|  |  | ACGTG | 2064 | 5 | - | Arabidopsis thaliana |
| PP2C | LOC105048609 | CGCACGTGTC | 2779 | 10 | + | Hordeum vulgare |
|  |  | CACGTG | 2781 | 6 | - | Arabidopsis thaliana |
|  |  | ACGTG | 2782 | 5 | + | Arabidopsis thaliana |
| SnRK | LOC105058890 | ACGTG | 2520 | 5 | + | Arabidopsis thaliana |
| ABI5 | LOC105034225 | CACGTG | 118 | 6 | + | Arabidopsis thaliana |
|  |  | ACGTG | 119 | 5 | + | Arabidopsis thaliana |
|  |  | CGCACGTGTC | 176 | 9 | + | Hordeum vulgare |
|  |  | CACGTG | 178 | 6 | + | Arabidopsis thaliana |
|  |  | ACGTG | 179 | 5 | + | Arabidopsis thaliana |
|  |  | ACGTG | 1173 | 5 | + | Arabidopsis thaliana |
|  |  | ACGTG | 2187 | 5 | + | Arabidopsis thaliana |
|  |  | AACCCGG | 2437 | 7 | - | Arabidopsis thaliana |
| WRI1 | LOC105046119 | ACGTG | 1121 | 5 | - | Arabidopsis thaliana |
| AP2-EREBP | LOC105041997 | ACGTG | 155 | 5 | - | Arabidopsis thaliana |
|  |  | ACGTG | 566 | 5 | - | Arabidopsis thaliana |
|  |  | GACACGTACGT | 581 | 10 | + | Oryza sativa |
|  |  | GACACGTACGT | 584 | 10 | - | Oryza sativa |
|  |  | ACGTG | 588 | 5 | + | Arabidopsis thaliana |
| bZIP | LOC105035446 | CACGTG | 283 | 6 | + | Arabidopsis thaliana |
|  |  | ACGTG | 284 | 5 | + | Arabidopsis thaliana |
|  |  | ACGTG | 1589 | 5 | - | Arabidopsis thaliana |
| C2C2-Dof | LOC105052365 | ACGTG | 120 | 5 | - | Arabidopsis thaliana |
|  |  | GACACGTGGC | 984 | 9 | + | Triticum aestivum |
|  |  | CACGTG | 986 | 6 | + | Arabidopsis thaliana |
|  |  | ACGTG | 987 | 5 | + | Arabidopsis thaliana |
|  |  | CACGTG | 1021 | 6 | + | Arabidopsis thaliana |
|  |  | ACGTG | 1022 | 5 | + | Arabidopsis thaliana |
|  |  | CGCACGTGTC | 1072 | 10 | - | Hordeum vulgare |
|  |  | CACGTG | 1074 | 6 | + | Arabidopsis thaliana |
|  |  | ACGTG | 1075 | 5 | + | Arabidopsis thaliana |
|  |  | ACGTG | 1340 | 5 | + | Arabidopsis thaliana |
| MYB | LOC105050171 | CACGTG | 252 | 6 | + | Arabidopsis thaliana |
|  |  | ACGTG | 253 | 5 | + | Arabidopsis thaliana |
|  |  | CACGTG | 284 | 6 | + | Arabidopsis thaliana |
|  |  | ACGTG | 285 | 5 | + | Arabidopsis thaliana |
|  |  | CACGTG | 627 | 6 | + | Arabidopsis thaliana |
|  |  | ACGTG | 628 | 5 | + | Arabidopsis thaliana |
|  |  | ACGTG | 943 | 5 | - | Arabidopsis thaliana |
|  |  | ACGTG | 1147 | 5 | + | Arabidopsis thaliana |
|  |  | ACGTG | 2105 | 5 | - | Arabidopsis thaliana |
|  |  | ACGTG | 2304 | 5 | + | Arabidopsis thaliana |
| NAC | LOC105044790 | ACGTG | 2249 | 5 | - | Arabidopsis thaliana |
|  |  | CACGTG | 2265 | 6 | - | Arabidopsis thaliana |
|  |  | ACGTG | 2266 | 5 | + | Arabidopsis thaliana |
